# IgT and IgD dominance in the teleost central nervous system

**DOI:** 10.3389/fimmu.2025.1657738

**Published:** 2025-08-29

**Authors:** Samuel Vicente-Gil, Valeria Pianese, Esther Morel, Beatriz Abós, Pablo Jiménez-Barrios, Pedro Perdiguero, Diana Martín, Rocío Simón, Simona Picchietti, Giuseppe Scapigliati, Carolina Tafalla

**Affiliations:** ^1^ Fish Immunology and Pathology Group, Biotechnology Department, National Institute for Agricultural and Food Research and Technology (INIA), Spanish Research Council (CSIC), Madrid, Spain; ^2^ Department for Innovation in Biological, Agro-food and Forest systems (DIBAF), University of Tuscia, Viterbo, Italy; ^3^ Department of Genetics, Physiology and Microbiology, Faculty of Biological Sciences, Complutense University of Madrid, Madrid, Spain; ^4^ Animal Health Research Center (CISA), National Institute for Agricultural and Food Research and Technology (INIA), Spanish Research Council (CSIC), Madrid, Spain

**Keywords:** teleost, central nervous system, B cells, IgT, IgD, rainbow trout

## Abstract

Although initially described as an immune-privileged site, the central nervous system (CNS) is now known to harbor a resident immune system, composed of different innate and adaptive cell types that maintain tissue homeostasis, are regulated in pathological conditions or brain injury and are in connection with the peripheral immune system. However, information regarding a characterization of the B cell subsets colonizing the CNS or their precise role is very scarce in mammals and even more in fish. Teleost fish exclusively express IgM, IgD and the teleost-specific IgT and their expression patterns in B cells define diverse B cell subsets. IgM is the main Ig isotype in systemic responses, while previous evidence suggested a mucosally dedicated role for IgT. Recent work by our group and others, also pointed to a role for IgD in maintaining mucosal homeostasis. However, in the current study, we demonstrate through different techniques that IgD^+^ and IgT^+^ expressing B cells are predominant in the teleost CNS, albeit a very different distribution. Additionally, B cell receptor (BCR) repertoire analysis pointed to both populations as antigen-experienced. Further research is needed to fully elucidate the specific roles of IgT and IgD in the teleost brain, but this discovery opens exciting avenues for future investigations in the fields of immunology and neuroscience.

## 1 Introduction

For many years, the central nervous system (CNS) has been considered an immune-privileged site, isolated from the peripheral immune system due to the lack of lymphatic vessels and through the action of the blood brain barrier (BBB) (1). However, this definition has been challenged in the last few years after the identification of a wide range of resident immune cells throughout the mammalian CNS. These resident immune cells include innate leukocyte populations such as natural killer (NK) cells (2), dendritic cells (DCs) and granulocytes (3) and also cells of the adaptive immune system such as T and B cells (3, 4). These studies point to the CNS as an immune-competent site that maintains active immune tolerance, performs tissue surveillance and establishes a bi-directional relation with the peripheral immune system (5).

A very high percentage of immune cells within the cerebrospinal fluid of healthy individuals correspond to T cells (6), and thus, the regulation and functionality of T cells in the mammalian CNS in homeostasis and pathogenic conditions has been the focus of many studies in the past years (7, 8). Although present in small numbers in the brain parenchyma in healthy individuals, T cells infiltrate the CNS from the periphery in many pathogenic models (8), including brain injury (9) or infections (5, 8). These recruited cells include both CD4^+^ and CD8^+^ T cell subsets (10).

Although very rare in the brain parenchyma, B cells have been reported in higher numbers in the CNS meninges and the cerebrospinal fluid (CSF) (11). Remarkably, a recent study has demonstrated that meningeal B cells originate from the skull bone marrow, while on the other hand, blood-derived antigen-experienced B cells continuously infiltrate the meninges with age (12). Nonetheless, B cells increase in the brain parenchyma in response to some infections such as human immunodeficiency virus (HIV) (13) or in pathological conditions such as multiple sclerosis (MS) (14, 15). In fact, a great number of studies have recently addressed the role of B cells in MS, a chronic inflammatory demyelinating disease of the CNS in humans, initially thought to be exclusively mediated by brain T cells. Multiple studies have now demonstrated that B cell presence and unbalance in the CNS are responsible for the local inflammation and in great part for the pathogenesis of the disease (14, 15). Interestingly, the detection of certain specific IgG antibodies in the CNS (which are not detected in blood) is one of the most consistent diagnostic features in MS (16). These B cells detected throughout the CNS of MS patients are mostly activated cells, including memory B cells, plasmablasts and plasma cells that produce either IgM or IgG (17). Interestingly, IgA, which is mainly produced in mammalian mucosal surfaces has been shown to act as a mediator between the brain and the gut during MS (18). Hence, these IgA-secreting plasma cells that recognize commensal antigens and that have been instructed in the gut, have been shown to travel to the CNS during MS (18). Additionally, IgA^+^ plasma cells educated in the gut have been shown to colonize the meninges to defend the brain also in homeostasis both in humans and mice (19). In the later, disturbance of the intestinal barrier by induced mucosal inflammation provokes a significant increase in the number of meningeal IgM and IgA-secreting plasmablasts (19).

To date, there is scarce knowledge regarding the immune response of the brain and the immune elements that are contained within the CNS in teleost fish species. Most of the studies performed up to this moment have mainly used immunohistochemical and transcriptional techniques to study the response to pathogens known to enter the CNS. For example, viral nervous necrosis virus (VNNV) is one of these fish pathogens with a strong tropism for the brain in species such as seabream (*Sparus aurata*) and sea bass (*Dicentrarchus labrax*) (20). Therefore, a few studies have investigated the immune response of the brain to this virus in these species, studying how immune genes were regulated (21) or how the pathogen was distributed (22). Recently, the presence of T cells in the sea bass brain parenchyma was demonstrated through different techniques including immunohistochemistry, electron microscopy and flow cytometry, suggesting a higher infiltrating capacity of fish T cells (23). These T cells seemed to correspond to both CD4^+^ and CD8^+^ cells, as revealed by transcriptomic analysis (23). Interestingly, in response to VNNV infection, T cells in the CNS of sea bass dramatically increased (23). Similarly, there is very little information regarding B cell populations in the teleost CNS. Transcriptional analyses have determined the up-regulation of IgM in the brain in response to nodavirus in sea bass (21), seabream (24) and Atlantic cod (*Gadus morhua*) (25). In seabream, an infiltration of IgM^+^ B cells in the brain in response to the virus was also demonstrated by immunohistochemistry (24). Similarly, IgM mRNA and protein levels were shown to increase in Patagonian blennie (*Eleginops maclovinus*) after *Francisella noatunensis* infection (26).

In addition to IgM, teleost fish express two other Ig isotypes, namely IgD and IgT, while lacking switched Ig isotypes present in mammals such as IgA, IgE or IgG (27). IgD in fish, as in mammals, is co-expressed on the cell surface of mature naïve B cells (28, 29). Upon antigen encounter, these B cells which have a high infiltrating capacity and are known to be one of the early responders to inflammation in fish, start a differentiation process towards IgM-secreting plasmablasts and eventually plasma cells, responsible for the secretion of high amounts of IgM, in a process in which they lose surface IgD (29). Additionally, cells exclusively expressing IgD (IgD^+^IgM^-^ B cells) have been identified in the blood of catfish (30) and in rainbow trout, mainly in mucosal surfaces such as gills, skin or intestine (31–33). These cells have a transcriptional profile of plasma-like cells (33) and they secrete IgD which can interact with the microbiota (32). Although the functionality of these cells and that of secreted IgD are still not well understood in fish nor mammals (34, 35), these IgD-secreting plasma-like cells have been proposed to play a role in mucosal homeostasis (32).

IgT is an Ig isotype only found in most (but not all) teleost fish species (27). Interestingly, in fish, V gene rearrangement to D and J regions within the IgH loci occurs independently for IgM/D and IgT, as each of these IgH genes is preceded by isotype-specific D and J gene segments (27). Therefore, VDJ recombination in fish results in the generation of a distinct B cell lineage expressing IgT, which is apparently independent of the IgM/D lineage. Because IgT cells were more abundant in mucosal surfaces than in systemic compartments and IgT responses were predominant in response to some mucosal parasites (36–38), IgT was initially considered a mucosally-dedicated Ig. However, this categorization does not seem so strict nowadays, since systemic IgT responses (39, 40) and mucosal responses that do not involve the up-regulation of IgT (41, 42) have also been revealed.

In this context, in the current study, we have investigated the B cell populations present in the teleost brain in homeostasis using adult rainbow trout (*Oncorhunchus mykiss*) as a model. For this, we have first investigated the distribution of IgM, IgD and IgT by immunohistochemistry in different sections of the rainbow trout CNS. Additionally, we have optimized a protocol to isolate leukocytes from the brain and study the different B cell subsets by flow cytometry and confocal microscopy, comparing them with blood B cells. Finally, we have carried out a repertoire analysis by next generation sequencing (NGS) to decipher how the variable regions (V_H_) of IgM, IgD and IgT heavy chains are distributed in this organ. Our work reveals a predominant presence of IgT^+^ and IgD^+^ B cell populations that follow distinct distributions within the teleost CNS implying different functionalities. This work constitutes the first description of B cell populations in the teleost brain that suggests previously unrecognized roles for these two still faintly characterized Igs. The results shown challenge existing dogmas about Ig distribution in fish and provide new insights into brain immunity.

## 2 Methods

### 2.1 Fish

Rainbow trout (*O. mykiss*) with a mean body weight of approximately 100 g were obtained from the *Cifuentes* fish farm (Cifuentes, Guadalajara, Spain). Fish were maintained at the animal facilities of the Animal Health Research Centre (CISA-INIA-CSIC) in an aerated recirculating system at 15°C, with a photoperiod of 12:12 h light/dark. Fish were fed once a day with a commercial diet (Skretting, Norway) and acclimatized to laboratory conditions for at least two weeks prior to sampling. During this period, no mortalities were experienced and no pathological signs were ever observed. Ammonia and nitrite levels were measured daily and maintained below 0.025 and 0.3 mg/l, respectively.

### 2.2 Immunohistochemistry

Fish were sacrificed by benzocaine (Sigma) overdose using a water bath with approximately 150 ppm of the anaesthetic following the recommendations of Zahl et al. (43) and the CNS including the hypothalamus and the pituitary carefully dissected. Rainbow trout brains were fixed in Bouin solution for 24 h at room temperature (RT) and then embedded in paraffin. Subsequently, 3.5 μm-thick tissue sections were mounted on Superfrost Plus slides (MenzelGläser). Endogenous peroxidase was quenched with Bloxall blocking solution (Palex) and antigens retrieved by heating in Tris–EDTA buffer (10 mM Tris base, 1 mM EDTA, pH 9) in a microwave oven for 5 min at 800 W and another 5 min at 450 W. Thereafter, non-specific binding was blocked with 5% bovine serum albumin (BSA) in Tris-buffered saline (TBS). Brain slides were then incubated with the different specific monoclonal antibodies (mAbs) against rainbow trout Igs. All these mAbs had been previously characterized and used for immunohistochemistry (44, 45). Anti-IgM was diluted 1:500, anti-trout IgT was diluted 1:50 and anti-trout IgD was used at 15 µg/ml, all in blocking solution (TBS with 5% BSA). The incubation with anti-trout IgM and anti-trout IgD was performed for 1 h at RT, whereas in the case of anti-trout IgT, the incubation was performed overnight (ON) at 4°C. In all cases, after the incubation, slides were washed and a secondary anti-mouse IgG mAb conjugated with horseradish peroxidase added. After repeated washing, the slides were incubated with IMPRESS KIT VR anti-mouse IgG (Palex). The specificity of the reactions was determined by omitting the primary antibodies. Sections were counterstained with Gill´s hematoxylin, dehydrated and mounted in DPX (di-N-butyl-phthalate in xylene). Images were acquired with a Leica DFC320 digital camera connected to a Leica DM LS optic microscope.

### 2.3 Leukocyte isolation

Fish were euthanized by benzocaine overdose and blood was extracted (approximately 1 ml) with a heparinized needle from the caudal vein and diluted 40 times with Leibovitz medium (L-15, Life Technologies) supplemented with 100 IU/ml penicillin, 100 μg/ml streptomycin (P/S), 10 units/ml heparin and 5% fetal calf serum (FCS) (all supplements also obtained from Life Technologies). Blood suspensions were layered onto 51% Percoll cushions and centrifuged at 400 x *g* for 30 min at 4°C, without brake. Leukocytes were collected from the interface and washed in L-15 containing P/S and 5% FCS. Brains were then sampled, after blood extraction, to avoid the contamination of samples with blood leukocytes. The tissues were then transferred into a tube containing L-15 supplemented with P/S, 2% FCS and 10 U/ml heparin, and cell suspensions were then obtained by pressing samples through a 100 μm nylon cell strainer (BD Biosciences). Cell suspensions were placed onto 30/51% discontinuous Percoll (GE Healthcare) density gradients and centrifuged at 400 x *g* for 30 min at 4°C, without brake. Cells at the interface, corresponding to leukocytes, were collected and washed in L-15 containing P/S and 5% FCS. In all cases, counting and cell viability were determined by trypan blue (Sigma) exclusion, mixing the 0.4% trypan blue solution with the cells at a 1:1 ratio.

### 2.4 Confocal microscopy

Brain and blood leukocytes isolated as described above were seeded on a poly-L-lysine (0.01% solution, Sigma-Aldrich)-coated slide and incubated at RT for 1 h in a humidified chamber. The cell samples were then fixed in 4% paraformaldehyde solution for 30 min at RT and subsequently incubated at RT for 1 h with blocking solution (TBS with 5% BSA and 0.5% saponin) in a humidified chamber. Unless otherwise specified, all the following incubation steps were performed for 1 h at RT in a humidified chamber. After the blocking step, samples were incubated with specific mouse mAbs recognizing the different Igs. Hence, samples were incubated individually with anti-trout IgM coupled to FITC (17 µg/ml), anti-trout IgD coupled to APC (50 μg/ml) or anti-trout IgT diluted 1:100 in blocking buffer. In the case of IgT, cells were subsequently stained with the secondary antibody anti-mouse IgG1 coupled to Alexa Fluor 488 (20 µg/ml). Samples incubated only with secondary anti-mouse IgG1 coupled to Alexa Fluor 488 and FITC or APC-conjugated mouse IgG1 isotypes were also included at the same concentrations (20, 17 and 50 μg/ml respectively) to confirm the specificity of the antibody signals. On the other hand, a double immunofluorescent detection of IgD and IgM was also performed. For this, the antibodies anti-trout IgM coupled to FITC (17 μg/ml) and anti-trout IgD coupled to APC (50 μg/ml) were used simultaneously on the cell samples. All the samples were counterstained with 1 μg/ml of DAPI (Sigma-Aldrich) for 10 min at RT, then rinsed with PBS 1x and mounted with Fluoromount (Sigma-Aldrich) for microscopy. Laser scanning confocal microscopy images were acquired with an inverted Zeiss Axiovert LSM 880 microscope with Zeiss Zen software. Tissue images were processed with Zeiss Zen and Adobe Photoshop CS6 software packages.

### 2.5 Flow cytometry

Brain and blood leukocytes isolated were also analyzed by flow cytometry. For this, cells were stained with anti-trout IgM [1.14 mAb mouse IgG1 coupled to R-phycoerythrin (R-PE); 0.25 μg/ml] (46) and anti-trout IgD [mAb mouse IgG1 coupled to allophycocyanin (APC); 5 μg/ml] (47) diluted in staining buffer (phenol red-free L-15 medium supplemented with 2% FCS) for 1 h in darkness at 4°C. Both antibodies were fluorescently labelled using R-PE or APC Lightning-Link labelling kits (Innova Biosciences) following the manufacturer’s instructions. Cells were also incubated in parallel with R-PE or APC-conjugated mouse IgG1 isotypes (clone MOPC-21, Biolegend) to confirm the specificity of the mAbs used. After staining, cells were washed twice in staining buffer and resuspended in staining buffer for their analysis in BD FACS Celesta with BD FACSDiva software (BD Biosciences). Doublets and dead cells were excluded from the flow cytometry analysis following the gating strategy described in **Supplementary Figure S4**. The data obtained was analyzed using the FlowJo^®^ v.10 software (FlowJo LLC, Tree Star). In all cases, cell viability was checked using 4-,6-diamine-20-phenylindole dihydrochlorid (DAPI) at 0.2 μg/ml.

### 2.6 ELISA

Brain and blood leukocytes (5x10^4^ cells) were seeded in 96-well plates and cultured for 48 h at 20°C in L-15 supplemented with P/S and 5% FCS. After this time, supernatants were collected to evaluate the concentration of IgM and IgD on the culture supernatants by ELISA. For this, 96 well-ELISA plates were coated overnight (ON) at 4°C with 100 μl of a mixture of anti-light chain (IgL) mAbs (2A1, 2H9, 2D12, 1B4, 3E4 and 1A6) (5 μg/ml) (48, 49) for detection of secreted IgD, or with 100 μl of anti-trout IgM (clone 1.14) (2 µg/ml) for detection of secreted IgM. In both cases, antibodies were diluted in 0.05 M carbonate buffer pH 9.7. Thereafter, three washing steps were carried out with PBS containing 0.05% Tween-20 (PBS-T) (Sigma-Aldrich) and non-specific binding sites were blocked with PBS containing 5% BSA for 1 h at RT. After three additional washing steps with PBS-T, 100 μl of the culture supernatants, diluted 1:2 in PBS supplemented with 1% BSA, were added to the wells and incubated for 1 h at RT. At this point, plates were washed with PBS-T and 100 µl of biotinylated anti-trout IgD or anti-trout IgM mAb added to the wells at a final concentration of 1 µg/ml in PBS with 1% BSA. Plates were then incubated for 1 h at RT. After three washing steps with PBS-T, the wells were incubated with 100 μl of Streptavidin-HRP (Thermo Fisher Scientific) at 100 ng/ml in PBS with 1% BSA for 1 h at RT. After another three washes, 100 μl of OPD (O-phenylenediamine Dihydrochloride) (Sigma) were added (1 mg/ml). The reaction was stopped by adding 50 μl of 2.5 M H_2_SO_4_ and absorbance at 490 nm measured in a FLUO Star Omega Microplate Reader (BMG Labtech). Positive and negative controls were included in all cases.

### 2.7 Western blot

Brain and blood leukocytes (5x10^4^ cells) were cultured for 48 h as described above. After this time, supernatants were collected to evaluate the concentration of secreted IgT by Western blot. For that, supernatants were mixed 1:2 with 2x loading buffer [10% glycerol, 10% 2-mercaptoethanol, 100 mM dithiothreitol (DTT), 4% SDS, 0.05% bromophenol blue and 0.125 M Tris] and heated at 90°C for 5 min. Proteins were then resolved by SDS-PAGE on a 15% acrylamide gel containing 6 M of urea. Secreted IgT was detected after electrotransfer to a 0.2 µm nitrocellulose membrane and incubation with the specific anti- IgT mAb (1:1000) ON at 4°C with continuous agitation. Thereafter, a secondary goat anti-mouse IgG-HRP (GE Healthcare) was added (1:5000) and incubated for 1 h at RT with continuous agitation. Visualization was carried out with a ChemiDoc XRS Gel Imagin System (Bio-Rad) and analyzed with the Image Lab Software (Bio-Rad).

### 2.8 Ig repertoire analysis

For the repertoire analysis total RNA was extracted from brain samples using TRI Reagent solution following the manufacturer´s instructions. An IgM, IgD and IgT repertoire analysis was performed in 6 brain samples. In all cases, RNA integrity was determined with a 2100 bioanalyzer using the Eukaryote Total RNA Nano assay (Agilent). Library construction was carried out using a custom protocol as follows. The cDNA synthesis from each sample was performed using 600 ng of total RNA in a reaction including an oligodT primer (**Supplementary Table S1**) and a template switch oligo (TSO) which included a unique molecular identifier (UMI) (**Supplementary Table S1**) that hybridizes to untemplated C nucleotides added by the reverse transcriptase during reverse transcription. The reaction was performed with SMARTScribe Reverse Transcriptase (Takara Bio) at 42°C for 90 minutes followed by incubation with 1 µl of uracil DNA glycosylase (5 U/µl, New England Biolabs) at 37°C for 40 min. The resultant cDNA was purified using NucleoSpin Gel and PCR Clean-up (Macherey-Nagel) at an elution volume of 20 µl. Target enrichment of 5’-end from IgM, IgD and IgT encoding transcripts were performed in two rounds of PCR followed by a third PCR for dual-indexation of PCR products, using in all reactions the Q5 High-Fidelity DNA Polymerase (New England Biolabs). The first PCR was performed with 1 µl of purified cDNA as template, the primer “Target_Enrichment_FW1” (**Supplementary Table S1**) partially complementary to a region of TSO sequence, and as reverse the primers R1 of each IgH “IgX_R1” (**Supplementary Table S1**), located in the constant region of the corresponding IgH, at a final concentration of 0.2 µM each. The PCR program was performed for 17 cycles (95°C for 10 s, 60°C for 20 s, and 72°C for 40 s) in a final volume reaction of 25 µl. PCR products were purified using NucleoSpin Gel and PCR Clean-up (Macherey-Nagel) at an elution volume of 25 µl. A second PCR was performed with 1 µl of purified PCR product as template, the primer “Target_Enrichment_FW2” (**Supplementary Table S1**) partially complementary to Target_Enrichment_FW1, and as reverse the primers R2 of each IgH “IgX_R2” located inner in the constant region of the corresponding IgH which include an additional tail corresponding with first part of P7 Illumina adaptor (**Supplementary Table S1**), both primers at a final concentration 0.2 µM. The PCR program was performed for 13 cycles (95°C for 10 s, 60°C for 20 s, and 72°C for 40 s) in a final volume reaction of 25 µl. PCR product was purified using NucleoSpin Gel and PCR Clean-up (Macherey-Nagel) at an elution volume of 25 µl. Finally, 1 µl of purified products from second PCR followed to a PCR round for indexing by which two different 8 nt sample-specific index, one in both side of amplicons, were incorporated together with the final region of P5 And P7 Illumina adaptors (**Supplementary Table S1**). PCR were performed with sample-specific primers (final concentration 0.2 µM each) in a final volume reaction of 25 µl. The PCR program was performed for 10 cycles (95°C for 30 s, 54°C for 30 s, and 72°C for 40 s). The PCR results were visualized in a 1.4% agarose gel to corroborate the positive results and the correct size of amplicons. Then, equal volume (10 µl) of PCR product from each sample was mixed together following with two steps of purification; the first one using NucleoSpin Gel and PCR Clean-up (Macherey-Nagel) with NTI buffer diluted 1/3 at an elution volume of 25 µl. This purified product was cleaned and size selected by using SPRIselect beads (Beckman Coulter) at a concentration of 0.6x. At this stage, the ready-to-sequencing library was checked using a tapestation with DNA 1,000 kit (Agilent), quantified with the Qubit ™ dsDNA HS Assay Kit (Invitrogen, Life Technologies) and sequenced with NextSeq™ 1000/2000 P1 Reagents (600 cycles) in a NextSeq1000 instrument (Illumina) with protocol 308:8:8:308 cycles and 30% of Phix.

Raw data consensus paired reads were merged using the PEAR software (50), with a minimum overlap size of 10 nt. These merged reads were demultiplexed using the FASTX-Barcode-Splitter tool from FASTX-Toolkit, allowing up to 5 mismatches and a partial overlap of 3. The first 20 nt from reverse primer used in the PCRs were used as barcodes for the identification of 3’ ends corresponding to the constant gene of each Ig. Then, all reads were grouped by their UMI sequences thanks to the MIGEC software (51). This tool also assembles the resulting grouped reads into unique sequences using only the first read as template and the collision filter on. UMI groups with less than 5 reads were discarded. As both IgM and IgD sequences shared the primer R2, absence of cross-assignment was checked using the UMI barcode. Finally, the assembled sequences were trimmed 14 nt at the beginning and 20 nt at the end to remove lower quality regions. The IgH sequences obtained were compared with the available information from *Oncorhynchus mykiss* contained in the international ImMunoGeneTics information system database (52) using the IMGT/HighV-QUEST tool v1.9.5 (53).

### 2.9 Statistical analysis

All data were analyzed and handled with GraphPad Software (GraphPad Prism v8.0.1, La Jolla California, USA). Prior to analysis, data were checked for normality using the Saphiro-Wilk test. An unpaired or paired two-tailed Student´s *t*-test was used in case of normally distributed data, whereas non-normal data was tested with the non-parametric Wilcoxon matched-pairs signed-rank test or the non-parametric Mann-Whitney test for unpaired data. To check the distribution of the CDR3 spectratyping, a Shapiro-Wilk test was performed. Data were presented as mean + SEM, and significance between means was stablished at *p* ≤ 0.05.

## 3 Results

### 3.1 Overview of Ig reactivity throughout the rainbow trout brain

**Figure 1:**
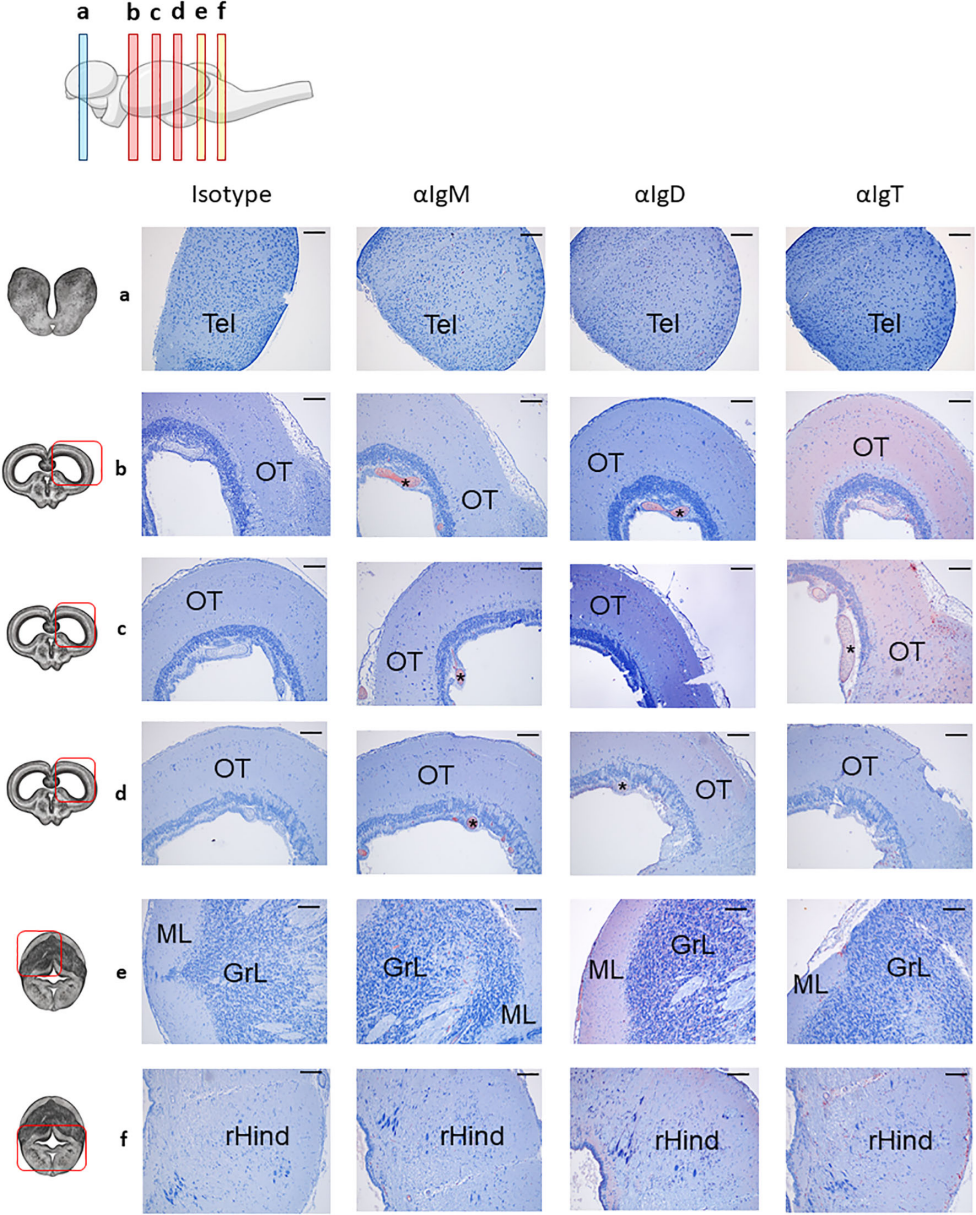
Distribution of B cell subsets throughout the rainbow trout brain. Rainbow trout brains were sampled and processed as described in the Material and Methods section for immunohistochemical analysis. A drawing of the rainbow trout brain indicating the location of the multiple areas analyzed is included (top) along with drawings of frontal views of the brain (left) corresponding to telencephalon (TEL) **(a)**, optic tectum (OT) **(b–d)** and cerebellum (Cb) **(e, f)**. The hindbrain (rHind) is clearly visualized in the lower part of the Cb sections **(f)**. Specific staining with anti-trout IgM, anti-trout IgD and anti-trout IgT mAbs, as well as isotype controls, are shown. (*) Blood vessels, molecular layer (ML), granular layer (GrL). Representative images from one fish are shown out of a total of 3 independent fish analyzed. Scale bars: 200 µm.

As in other vertebrates, the teleost brain is subdivided into five regions along the rostro-caudal and dorso-ventral axes: the telencephalon (Tel), the optic tectum (OT), the rest of the Forebrain/Midbrain (rForeMid; which includes the inferior lobe), the cerebellum (Cb) and the rest of the Hindbrain (rHind) (54). Hence, we investigated Ig reactivity throughout Tel, OT and Cb frontal sections in rainbow trout by immunohistochemistry. The Tel was the brain region with the lowest positivity for all three Igs (IgM, IgD, and IgT), showing only a few positive cells located inside small blood vessels (**Figure 1a**).

The OT is the largest region in the brain; consequently, we analyzed three different frontal sections corresponding to the anterior (b), medium (c) and posterior (d) OT to account for potential rostro-caudal heterogeneity. Across all OT areas, IgM reactivity was mainly found inside blood vessels (**Figure 1**, **Supplementary Figure S1**), whereas IgD and IgT exhibited significantly higher overall reactivity (**Figures 1b-d**). IgT^+^ cells were the dominant B cell population broadly distributed throughout the parenchyma, in both anterior (**Figure 1b**) and medium (**Figure 1c**) OT, along with high overall IgT reactivity suggesting some secreted IgT in these regions. Conversely, IgD^+^ cells were observed at significantly lower rates compared to IgT^+^ counterparts (**Figures 1b-d**). We also studied two different frontal sections of the Cb, corresponding to the most anterior (e) and posterior (f) sections. Within these sections, different Cb areas could be identified. In the upper part (**Figure 1e**), a molecular layer (ML) and a granular layer (GrL) were differentiated, whereas the bottom part (**Figure 1f**) was characterized by the presence of the rHind. In all these areas, IgM reactivity was consistently low, with positivity mostly confined to blood vessels (**Figures 1e, f**, **Supplementary Figure S1**). In contrast, both IgD^+^ and IgT^+^ cells were clearly identified within the cerebellar and the rHind parenchyma, with a pronounced predominance of IgD reactivity, reversing the OT’s IgT-dominant pattern (**Figures 1e, f**).

### 3.2 IgD reactivity in the rainbow trout optic tectum

**Figure 2:**
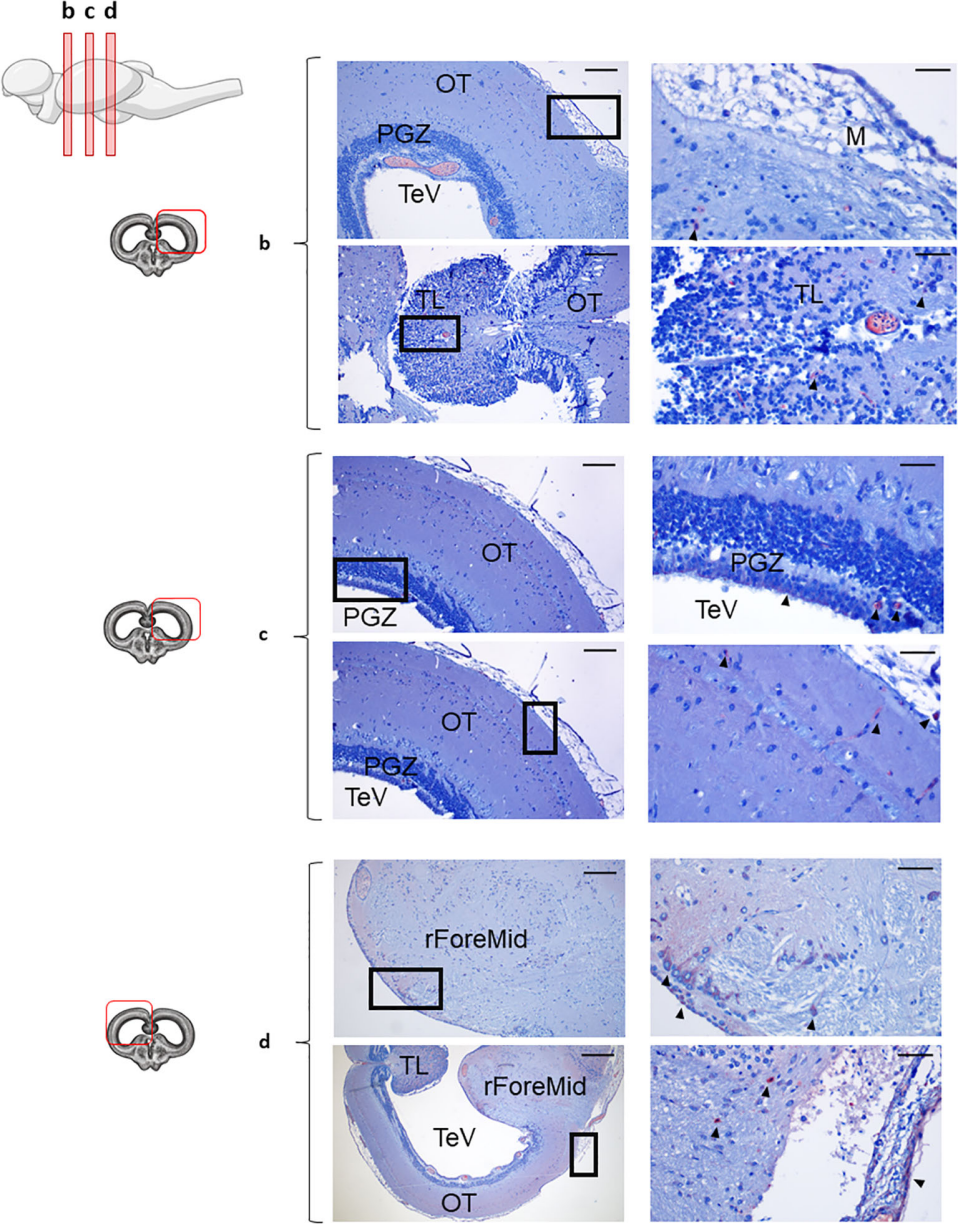
IgD^+^ B cells within the optic tectum. Rainbow trout brains were analyzed for immunohistochemical analysis of anterior **(b)**, medium **(c)** and posterior **(d)** regions of rainbow trout OT, using a specific anti-trout IgD mAb. Each panel displays representative images (left) of the different areas analyzed, with black squares indicating the areas shown at higher magnification (right). Arrowheads indicate IgD^+^ B cells. Isotype controls were included in all cases (not shown). A drawing of a rainbow trout brain indicating the location of the different areas examined is shown (top), along with drawings for each OT frontal view analyzed (left). (*) Blood vessels, primitive meninx (M), tectal ventricle (TeV), torus longitudinalis (TL), periventricular gray zone (PGZ), rest of the ForeMidbrain (rForeMid). Representative images from one fish are shown out of a total of 3 independent fish analyzed. Scale bars: 200 µm (left images) and 50 µm (right images) are indicated.

Given that B cells were mostly absent from the Tel brain area, we focused on studying in more detail the distribution of these cells in the OT and Cb. As mentioned above, some IgD reactivity was observed throughout the different sections of the OT, albeit at different frequencies (**Figures 2b–d**). Notably, IgD^+^ B cells were scarcely present in the anterior OT (**Figure 2b**), with only some IgD reactivity within the torus longitudinalis (TL) and the meninx, moderately abundant in the middle section (**Figure 2c**) and densely distributed in the most caudal region (**Figure 2d**). Especially in this caudal region, IgD^+^ B cells were clearly identified in the OT parenchyma as well as in the outermost layer of the OT, near the meninx, forming a barrier-like structure (**Figure 2d**). In the most posterior sections, IgD^+^ B cells were also clearly identified within the periventricular gray zone (PGZ) that englobes the tectal ventricle (TeV) (**Figure 2c**).

### 3.3 IgD reactivity in the rainbow trout cerebellum

**Figure 3:**
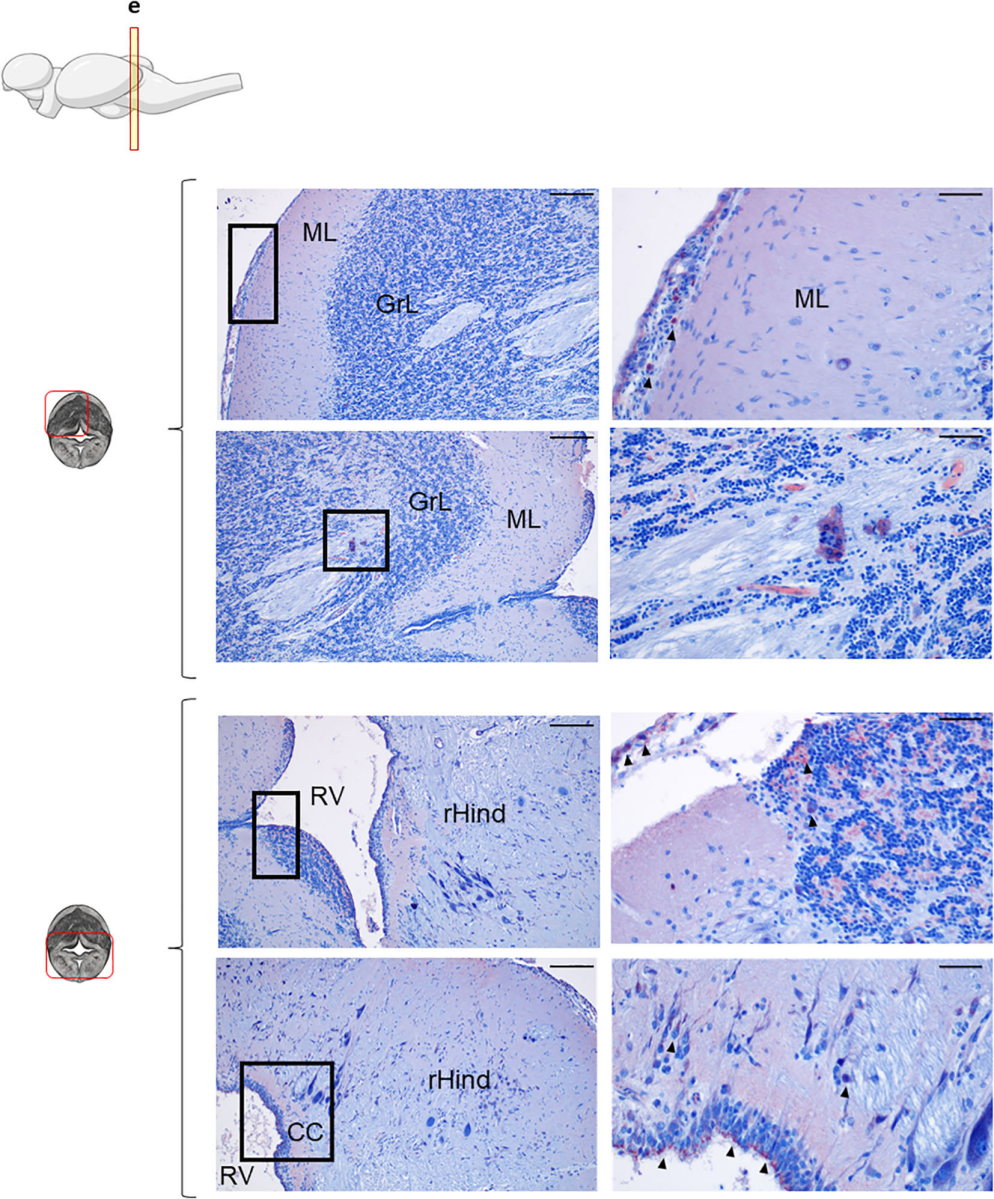
IgD^+^ B cells are mostly found in the cerebellum. Rainbow trout brains were analyzed for immunohistochemical analysis of the medium area of cerebellum (Cb) using a specific anti-trout IgD mAb. Each panel shows representative images of the different areas analyzed within the Cb section (left) with black squares indicating the areas shown at higher magnification (right). Arrowheads indicate IgD^+^ B cells. Isotypes controls were included in all cases (not shown). A drawing of a rainbow trout brain indicating the location of the section analyzed is included (top) along with drawings of the frontal views of each zone within analyzed Cb section. Molecular layer (ML), granular layer (GrL), rhomboencephalic ventricle (RV), rest of the hindbrain (rHind), crista cerebellaris (CC). Representative images from one fish are shown out of a total of 3 independent fish analyzed. Scale bars: 200 µm (left images) and 50 µm (right images) are indicated.

In the Cb, IgD^+^ B cells were analyzed across two different sections, an anterior and a more posterior section (**Figure 3**, **Supplementary Figure S2**, respectively). As mentioned above, within both sections, in the upper part, the ML and GrL were differentiated, whereas the bottom part of the sections corresponded to the rHind. In the most anterior section, IgD reactivity was clear in the meninx, with high amount of IgD^+^ B cells forming an external layer (**Figure 3**, upper panel). Although only a few IgD^+^ B cells were identified in the ML, there was an overall background staining in this area that suggested the presence of secreted IgD in the extracellular space (**Figure 3**, upper panel). In contrast, numerous IgD^+^ B cells were identified in the GrL, often in organized clusters in which IgD^+^ B cells surrounded other IgD^-^ B cells (**Figure 3**, upper panel). Interestingly, this cluster organization pattern was also observed in the bottom area of the Cb, where IgD^+^ cells appeared organized in a clusters in the crista cerebellaris (CC) (**Figure 3**, bottom panel). Additionally, IgD^+^ cells forming a barrier were also visualized in the rHind, surrounding the rhomboencephalic ventricle (RV) (**Figure 3**, bottom panel).

Less overall IgD positivity was observed in the more posterior section of the Cb analyzed (**Supplementary Figure S2**). In this cases, most of the IgD^+^ cells identified were found in the meninx forming a clear barrier that was more evident in the lower areas (**Supplementary Figure S2**). Additionally, some scattered IgD^+^ cells were visualized in the rHind parenchyma (**Supplementary Figure S2**, bottom panel).

### 3.4 IgT reactivity in the rainbow trout optic tectum

**Figure 4:**
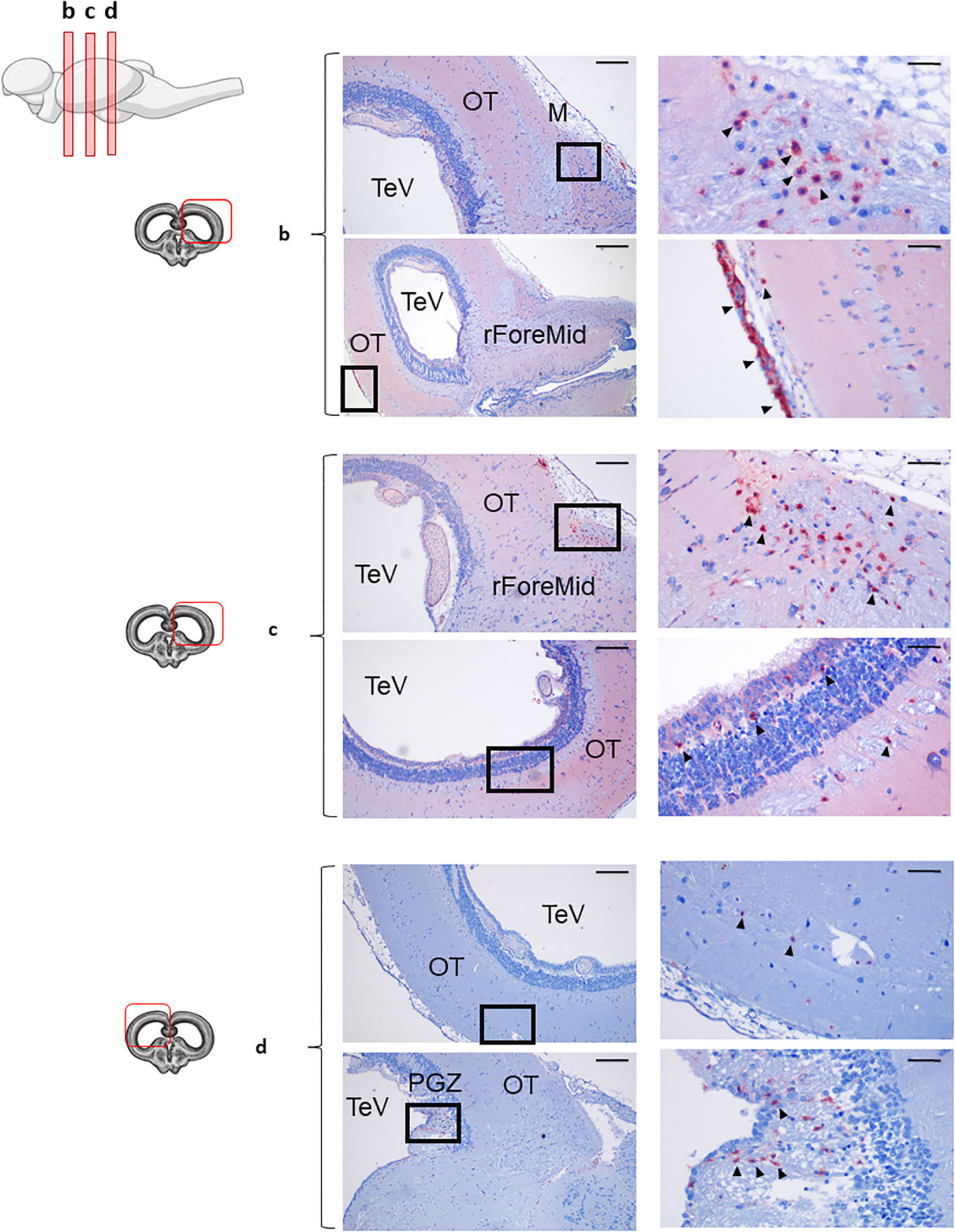
IgT^+^ B cells are abundant in the rainbow trout optic tectum. Rainbow trout brains were analyzed for immunohistochemical analysis of anterior **(b)**, medium **(c)** and posterior **(d)** regions of rainbow trout optic tectum (OT), using a specific anti-trout IgT mAb. Each panel displays representative images (left) of the different areas analyzed, with black squares indicating the areas shown at higher magnification (right). Arrowheads indicate IgT^+^ B cells. Isotypes controls were included in all cases (not shown). A drawing of a rainbow trout brain indicating the location of the different OT areas examined is included (top), along with drawings for each OT frontal view analyzed (left). Primitive meninx (M), tectal ventricle (TeV), ForeMidbrain (rForeMid) and periventricular gray zone (PGZ). Representative images from one fish are shown out of a total of 3 independent fish analyzed. Scale bars: 200 µm (left images) and 50 µm (right images) are indicated.

In the rainbow trout OT, IgT was the more predominant Ig expressed. Beyond IgT^+^ B cells, we observed extensive tissue-wide reactivity, especially in anterior and middle OT (**Figures 4b, c**). This intense staining pattern suggests IgT secretion into the extracellular spaces. In these most anterior regions of the OT, a high number of IgT^+^ B cells were distributed along the parenchyma (**Figures 4b, c**), but also associated to the meninx (**Figure 4b**). Notably, as described before for the IgD^+^ B cell population in the Cb, the population of IgT^+^ B cells identified in the external part of the meninx formed a dense barrier-like structure, as clearly visualized in (**Figure 4b**). In these anterior OT sections, IgT^+^ B cells were also identified in the most internal layers of the OT (**Figure 4c**), namely in the PGZ surrounding the TeV. In the posterior OT, the abundance of IgT^+^ B cells in the parenchyma decreased, whereas still some small groups of cells were found in the PGZ (**Figure 4d**).

### 3.5 IgT reactivity in the rainbow trout cerebellum

**Figure 5:**
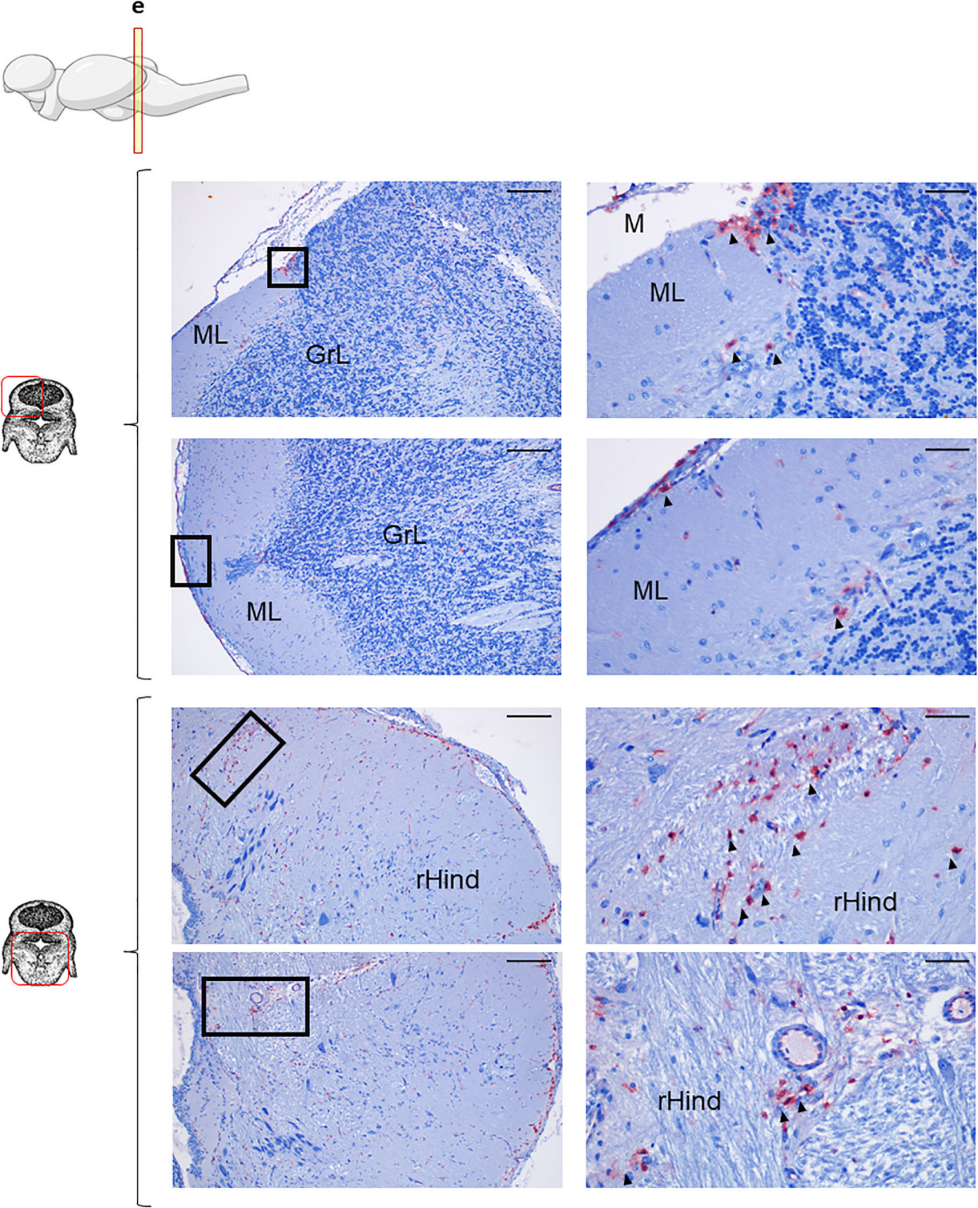
IgT^+^ B cells are also present in rainbow trout cerebellum. Rainbow trout brains were analyzed for immunohistochemical analysis of the medium area of cerebellum (Cb) using a specific anti-trout IgT mAb. Each panel shows representative images of the different areas analyzed within Cb section (left) with black squares indicating the areas shown at higher magnification (right). Arrowheads indicate IgT^+^ B cells. Isotypes controls were included in all cases (not shown). A drawing of a rainbow trout brain indicating the location of the analyzed area is included (top) along with drawings of the frontal views of each zone within analyzed Cb section. Molecular layer (ML), granular layer (GrL), meninx (M), rest of the hindbrain (rHind). Scale bars: 200 µm (left images) and 50 µm (right images) are indicated. Representative images from one fish are shown out of a total of 3 independent fish analyzed.

The overall IgT reactivity of the Cb was much lower than that of the OT (**Figure 5**), suggesting a lower amount of extracellular IgT in this tissue. Nonetheless, still numerous groups of IgT^+^ B cells could be clearly identified in the anterior part of Cb within both the GrL and the ML (**Figure 5**, upper panel). Some IgT^+^ cells were also found in the meninx layer, but in this case, their presence was more discontinuous than that observed in the OT (**Figure 5**, upper panel). In the bottom part of this anterior section of the Cb, high numbers of IgT^+^ B cells were distributed along the rHind parenchyma (**Figure 5**, bottom panel). When a more posterior section of the Cb was analyzed, a similar distribution of the IgT reactivity was observed in the rHind (**Supplementary Figure S3**).

### 3.6 Phenotypic analysis of B cells isolated from the rainbow trout brain

**Figure 6:**
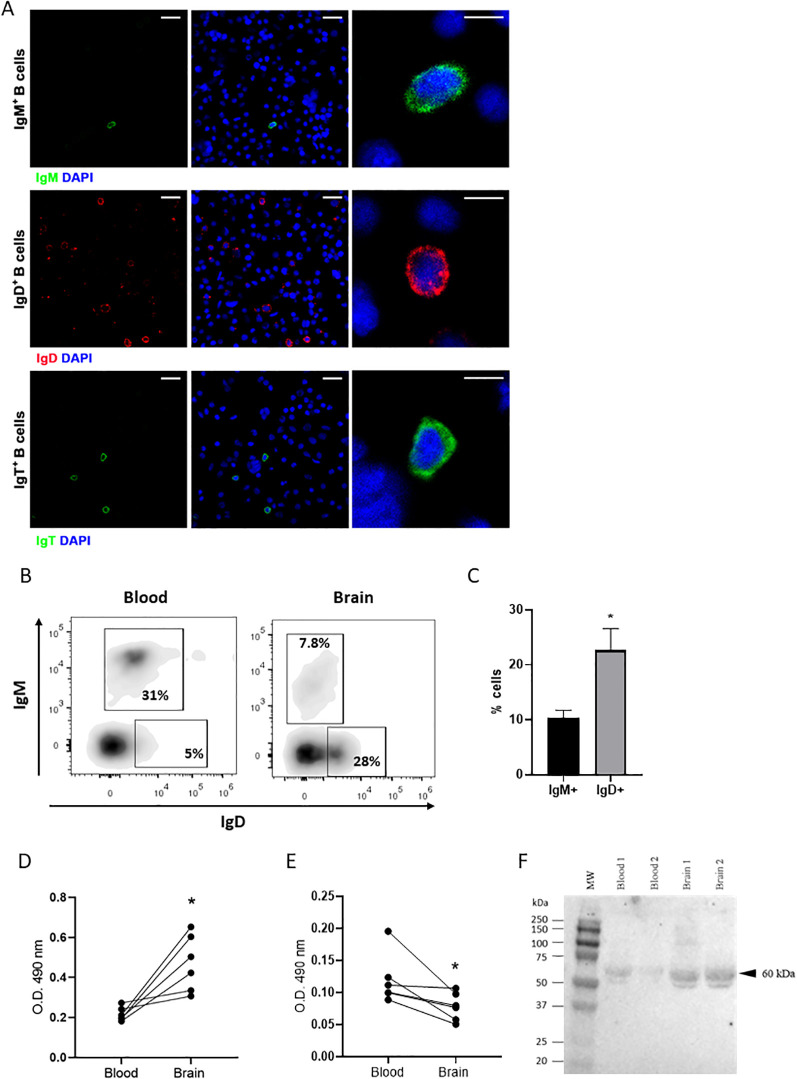
Phenotypic characterization of IgD^+^ and IgT^+^ B cells isolated from the rainbow trout brain. Brain and blood leukocytes were isolated as described in the Material and Methods section for a phenotypic characterization **(A)** Brain leukocytes were stained with specific mAbs for immunofluorescence analysis under the confocal microscope. All brain cell samples were counterstained with DAPI (1 μg/ml). Visualization of brain IgM^+^, IgD^+^ and IgT^+^ B cells are shown (left and middle panels) together with examples of each subset in digital magnification images (right panels). Scale bars: 20 μm (left and middle) and 5 μm (right). **(B)** Blood and brain leukocytes were stained with specific anti-trout IgM and anti-trout IgD mAbs. Representative dot plots show the percentages of IgM^+^ and IgD^+^ B cells in blood and brain leukocyte cultures isolated from a representative fish. **(C)** Graph shows the mean percentages of brain IgM^+^IgD^-^ and IgD^+^IgM^-^ B cells subsets (mean + SEM, n = 9 independent fish). **(D–F)** Isolated brain and blood leukocytes were cultured for 48 h at 20°C. After that time, supernatants were collected and the amount of secreted Igs were estimated by ELISA or Western blot. **(D, E)** Levels of secreted IgD **(D)** and IgM **(E)** were estimated by ELISA. Graphs show the absorbance at 490 nm obtained in the supernatants (n = 6 independent fish). **(F)** Secreted IgT (60 kDa) was estimated by Western blot. Statistical differences were evaluated by a paired two-tailed Student’s *t*-test when data were normally distributed, whereas non-normally distributed data were analyzed with the non-parametric Wilcoxon matched-pairs signed-rank test. Asterisks denote significant differences between different samples analysed (*p < 0.05).

To further investigate the phenotype of the B cells present in the rainbow trout brain, we performed an isolation of leukocyte populations by means of Percoll gradients. For this, complete brains, including all areas analyzed by immunohistochemistry, were dissected and used in the isolation procedure. The fish sampled were previously thoroughly bled (to reduce to a minimum blood contamination in brain samples). Within these total brain leukocyte populations, we could clearly identify B cells expressing the three different rainbow trout Ig isotypes by confocal microscopy (**Figure 6A**). In these cultures, IgD^+^ B cells were more abundant, followed by IgT^+^ B cells and lastly by IgM^+^ B cells (**Figure 6A**). These total brain leukocyte populations were also used to perform an analysis by flow cytometry, staining the cells with mAbs specific for IgM and IgD and using the gating strategy described in **Supplementary Figure S4**. In contrast to what occurs in blood, where most B cells expressing IgM co-express IgD on the cell surface (and are thus IgM^+^IgD^+^ B cells) (28), IgM^+^ B cells in the brain do not co-express IgD and are therefore IgM^+^IgD^-^ cells, such as those found in trout mucosal surfaces (33) (**Figures 6B, C**). In addition to these cells, brain leukocyte cultures contained a large population of cells exclusively expressing IgD on the cell surface (IgD^+^IgM^-^ B cells) (**Figures 6B, C**). This B cell subset accounted for up to 28% of all leukocytes in some fish, while it was barely present in systemic compartments, including blood (**Figure 6B**). The presence and prevalence of these different B cell subsets based on IgM and IgD surface expression was also confirmed by performing a double staining and examining the cells under the microscope. Through confocal microscopy, we could again confirm that IgD^+^IgM^-^ B cells were the most frequent B cell population within the IgM/D lineage in the rainbow trout brain (**Supplementary Figure S5**). We could not carry out a flow cytometry analysis of IgT^+^ B cells because the anti-IgT mAb we have available in our laboratory does not work in flow cytometry.

Cells that exclusively express IgM or IgD on the cell membrane are usually B cells that have started a differentiation program towards Ig-secreting cells, namely plasmablasts and eventually plasma cells (33). Hence, to investigate the Ig-secreting capacity of the brain B cell subsets, we cultured total brain leukocytes for 48 h and then collected the supernatants to determine the presence of secreted IgM or IgD by ELISA and of IgT through Western blot. We compared the results obtained to those obtained in supernatants derived from blood leukocyte cultures from the same fish. As observed in **Figure 6D**, the amount of secreted IgD observed in cultures from brain leukocytes was significantly higher than that of blood leukocytes. On the contrary, the amount of secreted IgM detected in brain leukocyte cultures was significantly lower than that detected on supernatants from blood leukocytes (**Figure 6E**). In the case of IgT, again the amount of secreted IgT observed in brain leukocyte cultures was significantly higher than that of blood leukocyte cultures (**Figure 6F**).

### 3.7 VDJ rearrangement in heavy chains

**Figure 7:**
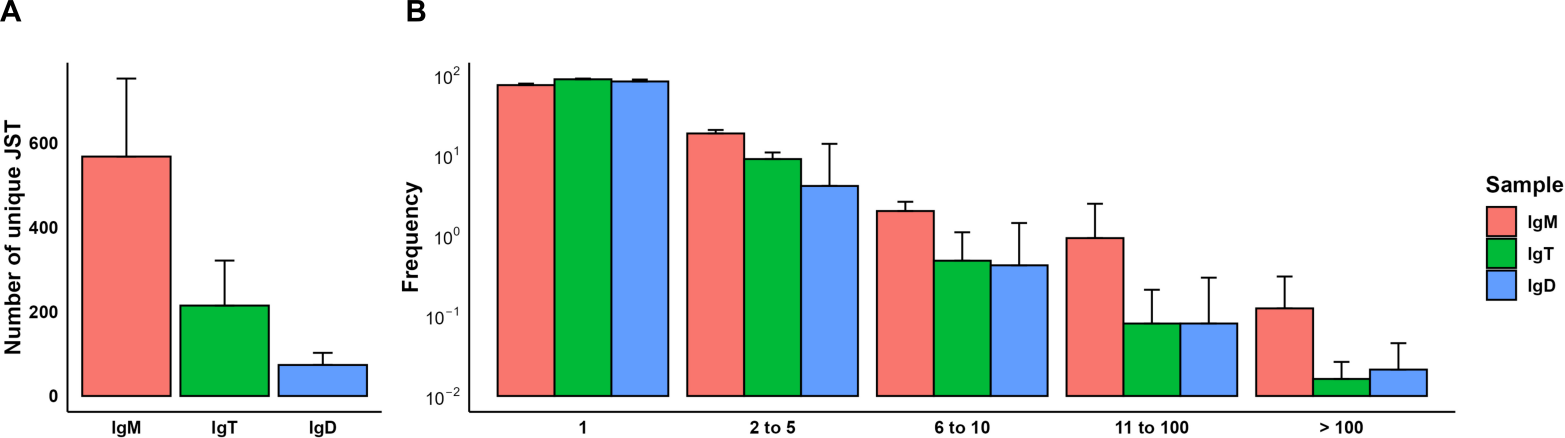
JST distribution in brain samples. IgH sequences were grouped by unique junction sequence types (JSTs), defined as a V_H_DJ_H_ rearrangement together with a specific CDR3 amino acid sequence. **(A)** Bar plot showing the number of unique JST split by heavy chain (mean + SD, n = 6). **(B)** Grouped clonal size distribution for the different heavy chains. Bar chart shows the relative frequency of JSTs observed n times in each chain (mean + SD, n = 6).

To provide us with a better understanding of the B cell receptor (BCR) diversity within the rainbow trout brain in homeostasis, we performed a repertoire analysis of the different Ig heavy chains (IgH) in RNA samples obtained from the CNS. First, IgH sequences for each Ig isotype were grouped into unique junction sequence types (JSTs), described as a V_H_DJ_H_ rearrangement together with a specific CDR3 amino acid sequence (55). In contrast to what is observed by immunohistochemistry or flow cytometry, the number of unique IgM sequences in the trout CNS significantly prevailed over the number of IgD and IgT sequences (**Figure 7A**). For all three Igs, we found sequences for which only one copy was obtained and also sequences that were repeated in variable numbers (**Figure 7B**). It is generally accepted that JSTs detected less than 3-5 times in a given tissue correspond to non-expanded antigen-naive B cells whereas JSTs detected more times reflect expanded antigen-experienced B cell clones, including antibody-secreting cells (39). Although the frequencies of expanded JSTs were similar for all three Igs, the frequency of IgM sequences repeated more than 11 times was slightly higher for IgM than for the other Igs (**Figure 7B**).

**Figure 8:**
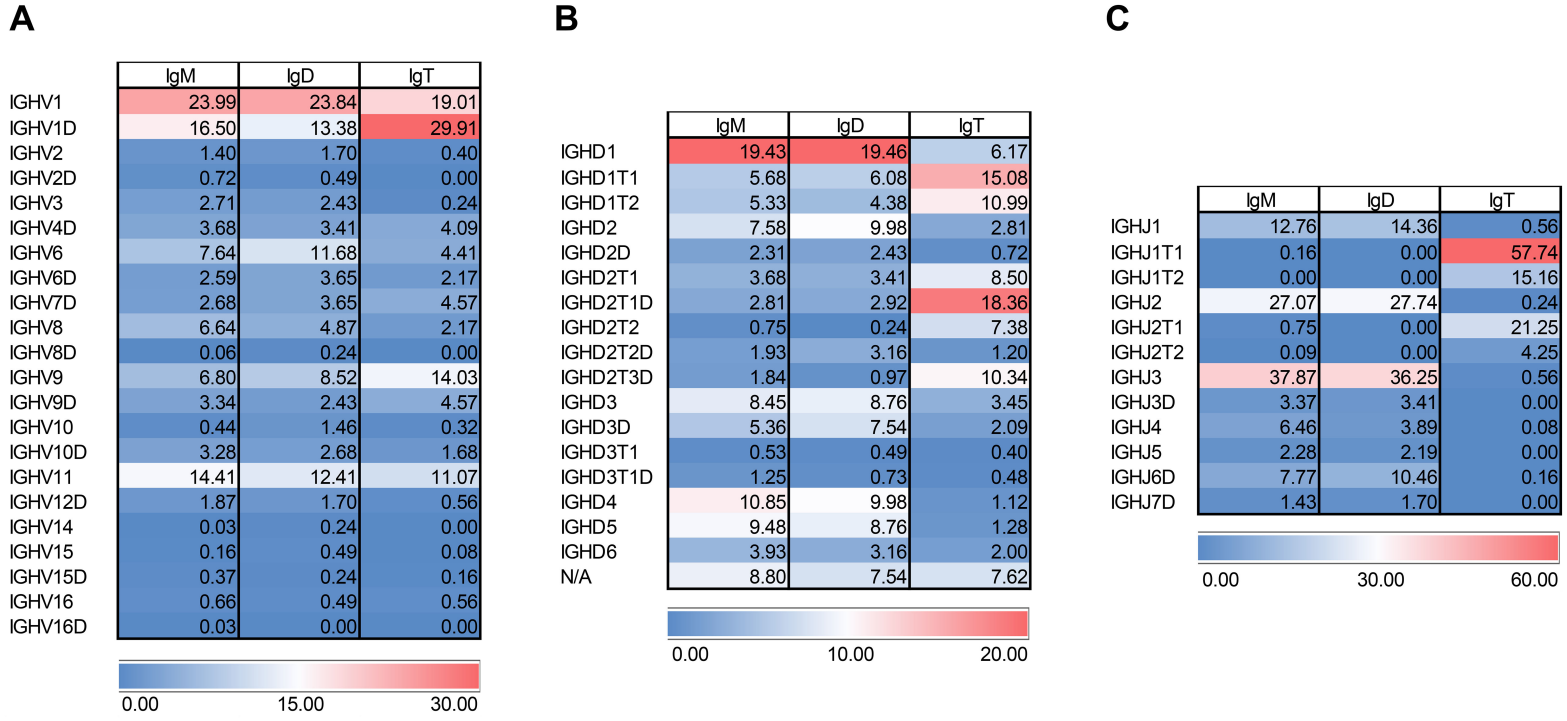
VHDJH gene configuration in brain samples across the different heavy chains. Heatmap representation of usage of **(A)** V_H_, **(B)** D_H_, and **(C)** J_H_ genes. Values are shown in percentage per IgH chain. Highest values are in red and lowest ones in blue.

To gain additional insight into the molecular configuration of Igs in the CNS, we dissected the heavy chain variable region (V_H_DJ_H_) family usage. Interestingly, in the CNS, the pattern of V_H_ gene usage was very similar for the three Igs. The three Ig isotypes preferentially used IGHV1 or its duplicated gene IGHV1D, followed by IGHV11 (**Figure 8A**). IGHV6 was also frequently used by IgM and IgD (~ 7.6% and 11.7%, respectively), but in this case, less used by IgT (~ 4.4%) (**Figure 8A**). On the contrary, IgT used IGHV9 at higher levels (~ 14%) than IgM and IgD (~ 6.8 and 8.5%) (**Figure 8A**).

In contrast, the pattern of D segment usage was more variable between IgT and that of IgM and IgD in the rainbow trout brain. In this case, IgM and IgD preferentially used IGHD1 followed by IGHD2, IGHD3, IGHD3D, IGHD4 and IGHD5, being all D segments close to the IgM/D constant regions (**Figure 8B**). IgT, on the other hand, preferentially used D segments usually associated with IgT such as IGHD1T1, IGHD1T2, IGHD2T1, IGHD2T1D, IGHD2T2, IGHD2T2D, IGHD2T3D (**Figure 8B**). Surprisingly, some of these D segments associated in the genome to IgT were also used to a moderate extent by IgM and IgD at variable percentages (from 0.2 to 6.1%) (**Figure 8B**). The same occurred with IgM/D-associated D genes which were mildly used by IgT (1.1 to 6.2%) (**Figure 8B**). Notably, a high percentage of sequences from all three Igs lacked a D segment (~ 8.8, 7.5 and 7.6 for IgM, IgD and IgT respectively).

A strong selection for defined J_H_ segments were found for Ig sequences in the brain. Hence, IgM and IgD used IGHJ3 at very high percentages (~ 37.8% and 36.2% for IgM and IgD respectively), followed by IGHJ2 (~ 27.1% and 27.7% for IgM and IgD respectively). Other J_H_ segments such as IGHJ1 and IGHJ6D were used by these two Igs at lower percentages (**Figure 8C**). In contrast, IgT sequences preferentially used IGHJ1T1 (~ 57.7%) followed by IGHJ2T2 (~ 21.3%) and IGHJ1T2 (~ 15.2%) (**Figure 8C**).

### 3.8 Clonal selection across the different heavy chains

**Figure 9:**
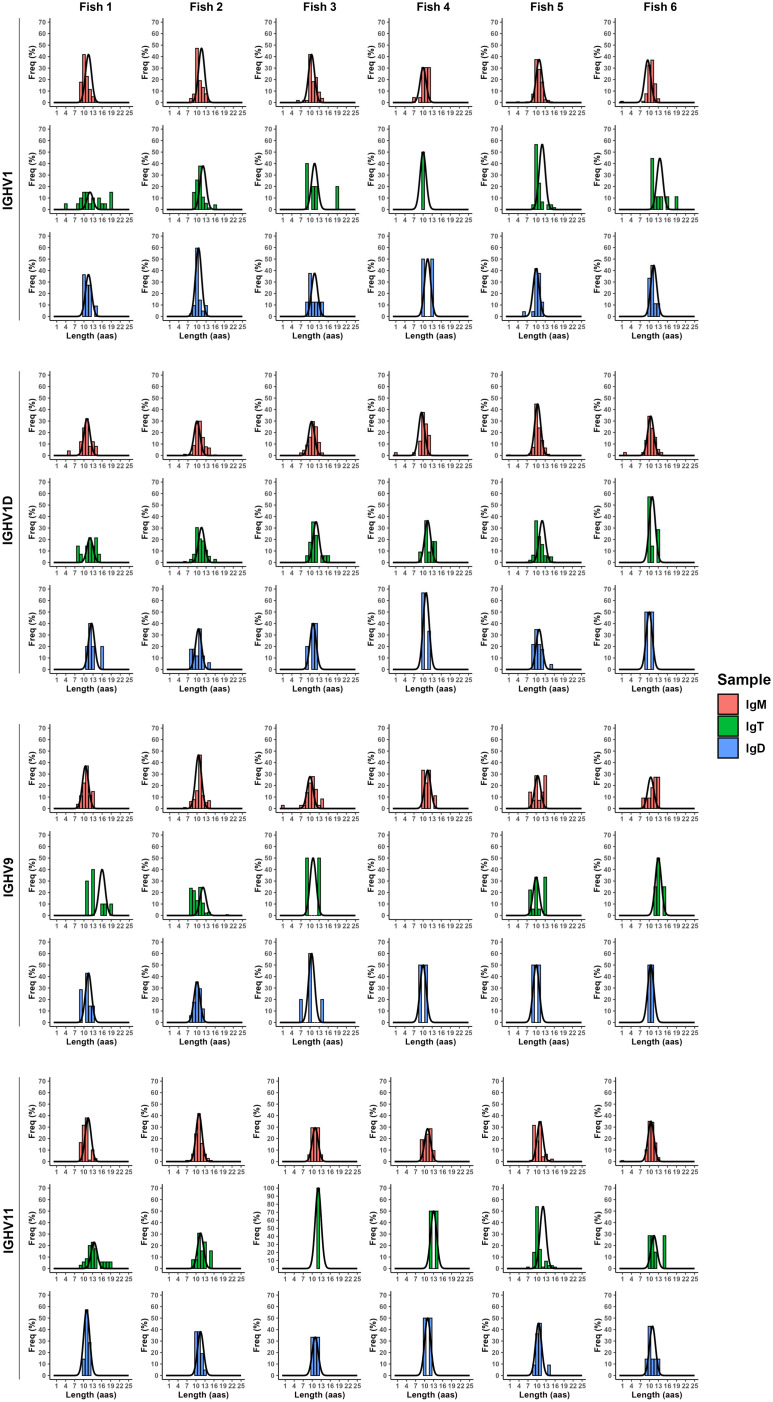
Clonal selection across the different IgH. CDR3 spectrotypes from the JSTs with the most used V_H_ genes: IGHV1, IGHV1D, IGHV9, and IGHV11. The black curved lines represent the theoretical normal distribution adjusted to each plot. All graphs follow a non-normal distribution, as tested by Shapiro’s assay.

To study the degree to which these B cells in the brain of non-immunized fish had experienced clonal selection, we also undertook a spectratyping analysis of the CDR3 regions of the different BCRs in which the distribution of BCR lengths is analyzed to provide with an indication of how BCR repertoire change (56). Hence, selections and expansions of B cells expressing a specific BCR with a defined CDR3 sequence lead to the modification of the Gaussian-like CDR3 length distribution that is found in physiological conditions (56). For each IgH, we first performed the analysis with all CDR3 sequences, not taking into account the V_H_ genes used. In this case, IgT CDR3 length distributions seemed to be the ones that differed more from a normal Gaussian-like distribution (**Supplementary Figure S6**). We then performed the CDR3 spectratyping analysis focusing on CDR3 sequences that used a defined V_H_ segment, namely IGHV1, IGHV1D, IGHV9, and IGHV11, those mostly used in the brain. In these analyses, it seemed more clear that IgM CDR3 length distributions were much closer to normality than those of IgD and IgT (**Figure 9**). For IgD, the highest selection seemed to occur when the IGHV9 was used, whereas for IgT, this happened when either the IGHV9 or the IGHV11 fragments were used (**Figure 9**). Important differences were found among the individual fish, with some fish having selected one or two CDR3 lengths for IgD or IgT (**Figure 9**).

## 4 Discussion

In the current study we have demonstrated through the use of different techniques that IgD- and IgT-expressing B cells are dominant over IgM-expressing cells in the rainbow trout brain, with cells positive for both Igs having been clearly identified throughout different regions of the CNS, both in association to the meninx and other superficial areas, but also clearly embedded within the brain parenchyma. Both IgT and IgD had been previously associated with mucosal responses in teleosts (and mammals in the case of IgD), but the data here presented suggest novel roles in a previously unrecognized target organ.

IgT was identified in 2005 as a teleost-specific Ig (27). The genomic distribution of the IgT locus, containing specific D and J gene segments for the generation of diversity, already anticipated that IgT expression was apparently independent of IgM and IgD (27), fact that was later confirmed with the generation of a specific mAb in rainbow trout (36). In rainbow trout, the ratio of IgT-expressing to IgM-expressing cells was shown to be higher in mucosal surfaces than in systemic compartments and IgT responses were revealed prevalent in response to parasites such as *Ceratomyxa shasta* (36) in the gut or *Ichthyophthirius multifiliis* in skin and gills (37, 38). For this reason, IgT was considered a mucosally-dedicated Ig in teleost and a functional analogue of mammalian IgA (57). Nonetheless, this mucosal distribution of IgT seems highly dependent on the fish species and for example in adult zebrafish IgT transcripts are exclusively found in primary immune organs, namely thymus and head kidney (58). Additionally, in the past years, examples of non-mucosal roles for IgT and mucosal responses in which IgT is not mainly involved are being reported in different species, revealing some flexibility. For example, IgT responses have been shown to be prevailing in the kidney of rainbow trout infected with the myxozoan *Tetracapsuloides bryosalmonae* during proliferative kidney disease (PKD) (40). Disturbance of IgT repertoires was also reported in rainbow trout infected by intraperitoneal injection with viral hemorrhagic septicemia virus (VHSV) (39). On the other hand, in red mark syndrome (RMS), a rainbow trout disease that provokes characteristic skin lesions, skin IgM and IgD responses are prevalent over IgT (41). In the current study, we have demonstrated that IgT^+^ B cells are present in the CNS of non-immunized adult rainbow trout, revealing a novel non-mucosa role for this teleost-specific Ig. Interestingly, in humans, IgA-secreting plasma cells instructed in the gut were found in the CNS of MS-affected patients (18). Hence, during MS, IgA^+^ B cells specific for defined microbiota strains in the gut, travel to the inflamed CNS and consequently, BCR repertoires are highly shared between gut and brain B cells during the disease (18). In homeostasis, IgA-expressing plasmablasts were also identified adjacent to the dural sinuses in both mice and human meninges (19). The presence of these cells increased with age and was again highly dependent on the intestinal microbiota, sharing a high percentage of B cell clones between both tissues (19). In fact, the complex communication network established between the gut and the brain is widely studied nowadays in humans, being responsible for the regulation of many behavioral and physiological body functions and commonly referred to as the gut-brain axis (59). In our work, we have determined whether BCR clonotypes obtained in the brain for IgT and other Igs were shared with those found in the intestine, finding very low shared percentages, sometimes close to cero (data not shown). This reveals that at least in non-immunized rainbow trout, the IgT^+^ B cell clones found in the CNS are completely independent of those in the intestine, ruling out these cell populations as mediators of the recognized connection between the brain and the gut (brain-gut axis). Furthermore, in the rainbow trout brain, although IgT^+^ B cells were identified in the meninx forming a barrier-like structure, especially in the OT region, these cells were also clearly visualized in parenchyma, throughout the OT, the Cb and the rHind. These cells were shown to have IgT secretory capacity, as demonstrated by Western blot analysis of the supernatants collected from brain leukocyte cultures, yet the specificity of this secreted IgT is something that warrants investigation in future studies.

The precise functionality of IgD is still enigmatic in fish as in mammals. Although it was initially thought that when co-expressed on the surface of naïve B cells, IgM and IgD BCRs were interchangeable (60, 61), posterior studies revealed some differences in their antigen affinity and down-stream signaling. For example, IgD deficiency seems to impair affinity maturation (62). Additionally, the fact that IgD has a longer hinge region than IgM renders it more flexible and reactive to polymeric antigens, whereas unresponsive to monomeric antigens (63). Even more intriguing is the role of IgD^+^IgM^-^ B cells which arise through an alternative form of class switch recombination (CSR) (64). These cells, which are highly clonal (suggesting division) and have highly mutated Ig V_H_ regions, have been shown to be often poly- and auto-reactive and therefore seem to play a relevant but not still well defined role in peripheral tolerance (65–67). Additionally, some of these IgD^+^IgM^−^ B cells, differentiate to plasmablasts and plasma cells that secrete IgD. The function of secreted IgD in humans is largely unknown but a relevant mucosal role has been hypothesized based on recent evidence such as the fact that IgM-to-IgD CSR occurs mainly in nasopharyngeal and other upper respiratory tract compartments (68). Interestingly, although these cells have not been located in the human intestinal tract, secreted IgD has been shown to coat the intestinal microbiota and found to be reactive to some food allergens (35, 69). Additionally, IgD has been shown to coat some respiratory pathogens (70), and to establish an Fc receptor-independent interaction with innate populations such as basophils, mast cells, monocytes and myeloid DCs (71). The fact that IgD-expressing plasmablasts were commonly found in rainbow trout mucosal surfaces where they interact with the microbiota, also suggested a role for this Ig in regulating mucosal responses and homeostasis in these species (32, 33). Nonetheless, in the current work, we have identified the brain as an alternative tissue in which IgD^+^ B cells are abundant. The fact that IgM staining was mostly confined to blood vessels in the tissue seemed to indicate that these cells found in the CNS parenchyma and the meninx were exclusively producing IgD. This was later confirmed analyzing the isolated leukocytes obtained from the CNS by means of confocal microscopy and flow cytometry. Furthermore, as the IgD^+^IgM^-^ B cell populations in the rainbow trout skin or gills (33), IgD^+^IgM^-^ B cells in the brain also had the capacity to secrete IgD. Again, the role of these cells and that of the secreted IgD in the CNS remains unknown at this point and will be addressed in future studies. Interestingly, intrathecal synthesis of IgD has been reported in the CNS of patients with different type of neurological diseases including MS or bacterial and viral meningitis (72). Posterior studies demonstrated that this secreted IgD was especially important when MS was in a clinically active relapsing phase (73). Yet again, in rainbow trout, we have demonstrated the presence of IgD^+^ B cells not only in the meninx layer but also in the CNS parenchyma. Remarkably, the distribution of IgD^+^ B cells throughout the CNS parenchyma was different than that of IgT^+^ B cells, suggesting differential roles for the two B cell subsets. IgD^+^ B cells were more predominant in the more posterior sections of the rainbow trout CNS, mainly found in the GrL of the Cb and also in the rHind, forming a barrier-like structure surrounding the RV. The specific functions of IgD^+^ B cells and secreted IgD in these CNS areas should be further investigated in future studies.

To further decipher the origin and functionality of these B cell populations in the rainbow trout CNS, we have performed a repertoire analysis to study BCR diversity. One of the things that stands out from these studies is the fact that IgM and IgD repertoires in the CNS have a very similar profile of V_H_, D and J_H_ segment usage. This suggests that the IgD^+^IgM^-^ B cells in the CNS have possibly developed from an IgM^+^IgD^+^ naïve B cell locally. While they share V_H_ segments, IgT often uses distinct D and J_H_ gene segments compared to IgM and IgD. This contributes to differences in the CDR3 region, which is crucial for antigen binding specificity (55). For this reason, the profile of VDH segment usage in IgT in the CNS was quite different to that of IgM and IgD, which was suspected given they constitute an independent lineage of cells. Nonetheless, it was surprising to see that some D segments thought to be exclusively used by IgT were also used by IgM and IgD to some extent (for example IGHD2T1) and vice versa. This implies that there are mechanisms for IgM, IgD and IgT to use genomic sequences (D gene segments) for rearrangement that are further apart in the genome (27). Whether this is specific for the CNS or something that can happen across tissues should be further confirmed. From the CDR3 spectratyping analysis, what we clearly see is that the IgD and IgT BCR sequences have experienced a higher degree of selection than IgM. This is especially evident when specific highly used V_H_ segments are selected. This together with the Ig secreting capacity demonstrated by means of ELISA (for IgD) and Western blot (for IgT) support the hypothesis that both IgT^+^ and IgD^+^ B cells in the rainbow trout CNS are antigen-experienced B cells that have started a differentiation towards plasmablasts/plasma cells. Whether these are exogenous antigens that cross the BBB or local endogenous antigens should be addressed in future studies. Finally, there seems to be a discordance between IgM mRNA and protein levels in the rainbow trout CNS, since although IgM^+^ B cells were rare in flow cytometry analysis, and seemed almost exclusively confined to blood vessels in IHC, the amount of IgM transcripts in CNS RNA samples is much higher than that of the other Igs. We have to rule out that this is due to contaminating blood in the samples because in that case, more IgM^+^IgD^+^ B cells would had been detected in flow cytometry analysis. Hence, it might be possible that IgM transcription levels do not exactly correlate with protein synthesis due to possible post-transcriptional regulations, known to take place for IgM (74, 75).

Although more and more B cells arise as fundamental players in the pathophysiology of autoimmune diseases of the CNS in mammals (14–19), there is still scarce information about the types of B cells present in this tissue and their behavior during homeostasis and disease. The data here presented in which different B cell populations have been identified throughout different regions of the CNS in a primitive vertebrate such as rainbow trout provides us with valuable comparative perspectives for understanding the evolution of neuroimmunology in vertebrates. Furthermore, the identification of IgD^+^ and IgT^+^ B cell populations in the teleost CNS challenges the conventional understanding of the tissue-specific distribution of Ig isotypes in fish, especially taking into account that these cells do not seem to be connected to intestinal B cell populations due to the lack of shared clonotypes. The data presented also reveals a more active and specialized immune environment in the brain than previously thought and raises important questions about the role that these Igs have in the CNS homeostasis. Our results, together with the recent discovery of a specialized microbiome in the brain of healthy fish (76), strongly suggests that fish have evolved specialized immune mechanisms within the CNS. How these specialized immune components contribute to its protection during pathology should be addressed in future studies determining how these B cell populations react to pathogens with a CNS tropism. These studies will be of relevance for future vaccine design and disease management in aquaculture.

## Data Availability

The data discussed in this publication has been deposited in NCBI’s BioProject database and is accessible through the BioProject accession number PRJNA1308505 (http://www.ncbi.nlm.nih.gov/bioproject/1308505).
