# Supplementary material for: IgT and IgD dominance in the teleost central nervous system
Source: Front Immunol. 2025 Aug 29;16:1657738. doi: 10.3389/fimmu.2025.1657738 (PMC12425738; doi:10.3389/fimmu.2025.1657738)
Supplement: Supplementary file 1 [file DataSheet1.pdf]

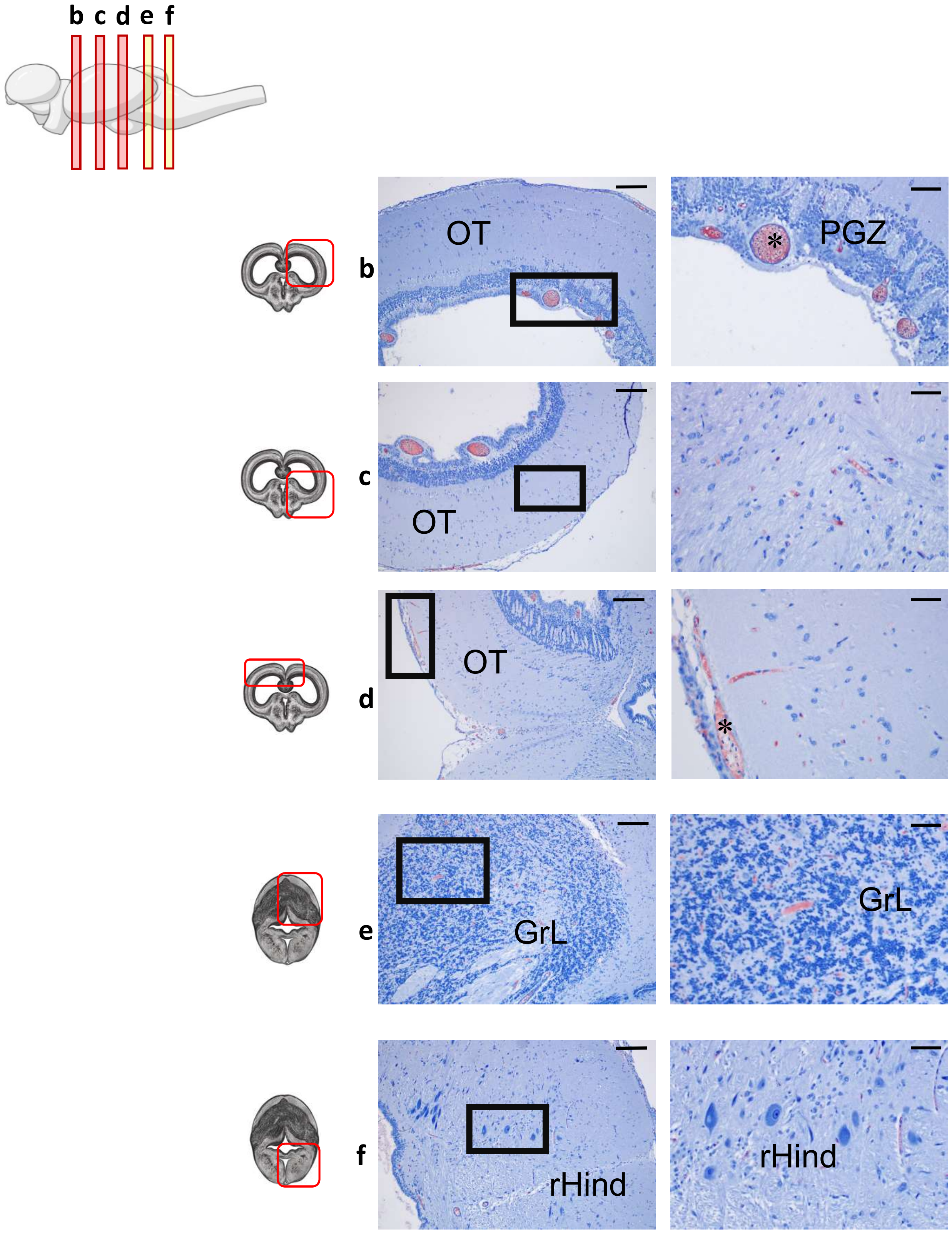

Figure S1. **IgM⁺ B cells are mainly located in vessels**. Rainbow trout brains were sampled and processed as described in the Material and Methods section for immunohistochemical analysis of the anterior (b), medium (c) and posterior (d) areas of optic tectum (OT) and medium (e) and posterior (f) areas of cerebellum (Cb) using a specific anti-trout IgM mAb. A drawing of a rainbow trout brain indicating the location of the sections analyzed is shown (top) along with drawings of the frontal views of each zone in the OT and Cb. Representative images of different zones analyzed are shown (left panels) with black squares indicating the areas shown at higher magnification (right panels). Isotypes controls were included in all cases (not shown). Periventricular gray zone (PGZ), granular layer (GrL), rest of the hindbrain (rHind). Scale bars: 200 µm (left images); 50 µm (right images).

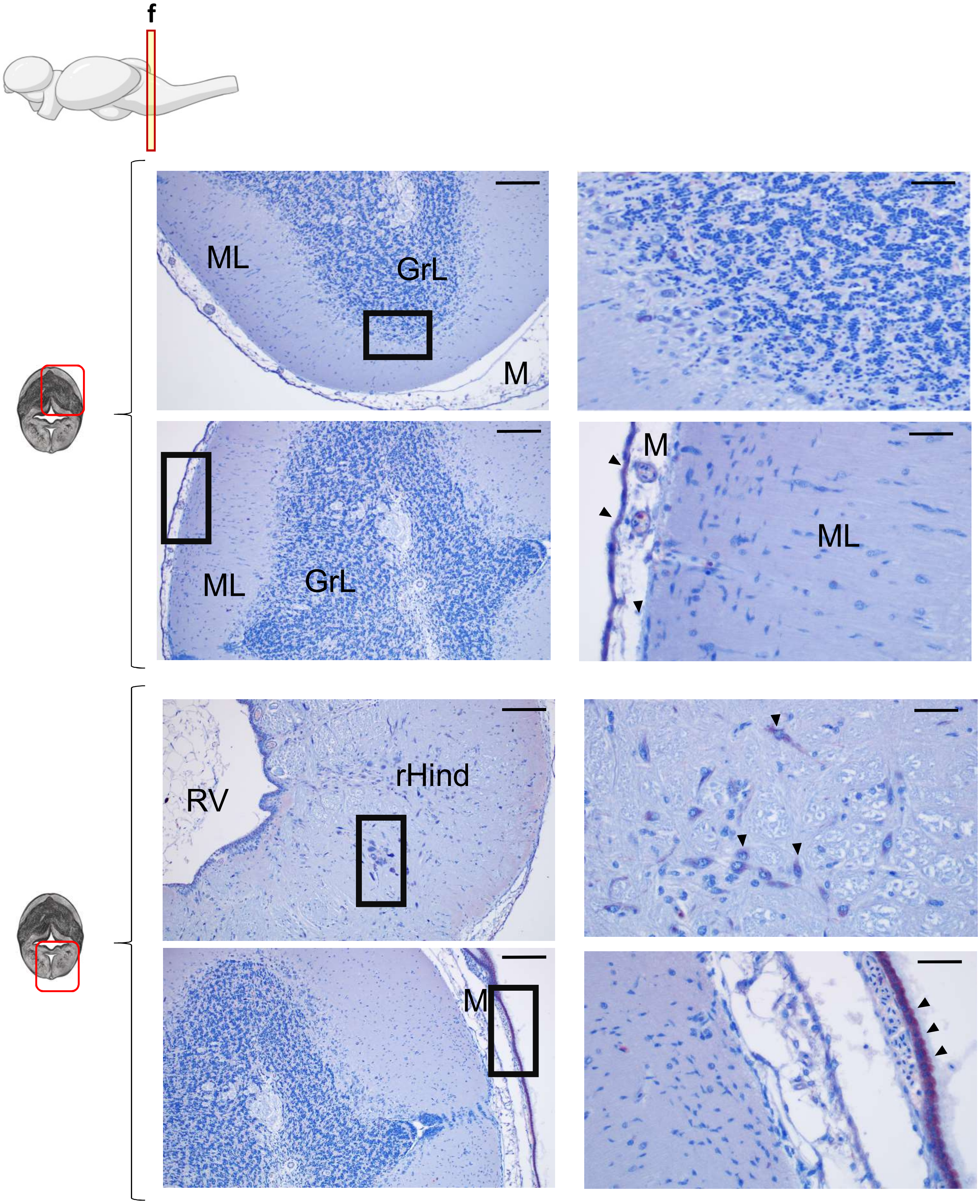

Figure S2. IgD$^+$ B cells are abundant in posterior part of rainbow trout cerebellum. Rainbow trout brains were sampled and processed as described in the Material and Methods section for immunohistochemical analysis of the posterior area of cerebellum (Cb) using a specific anti-trout IgD mAb. Representative images are shown (left panels) with black squares indicating the areas shown at higher magnification (right panels). Arrowheads indicate IgD$^+$ B cells. Isotypes controls were included in all cases (not shown). A drawing of a rainbow trout brain indicating the location of the section analyzed is shown (top) along with drawings of the frontal views of each zone within analyzed Cb section. Molecular layer (ML), granular layer (GrL), meninx (M), rhomboencephalic ventricle (RV), rest of hindbrain (rHind). Scales bars: 200 µm (left images); 50 µm (right images).

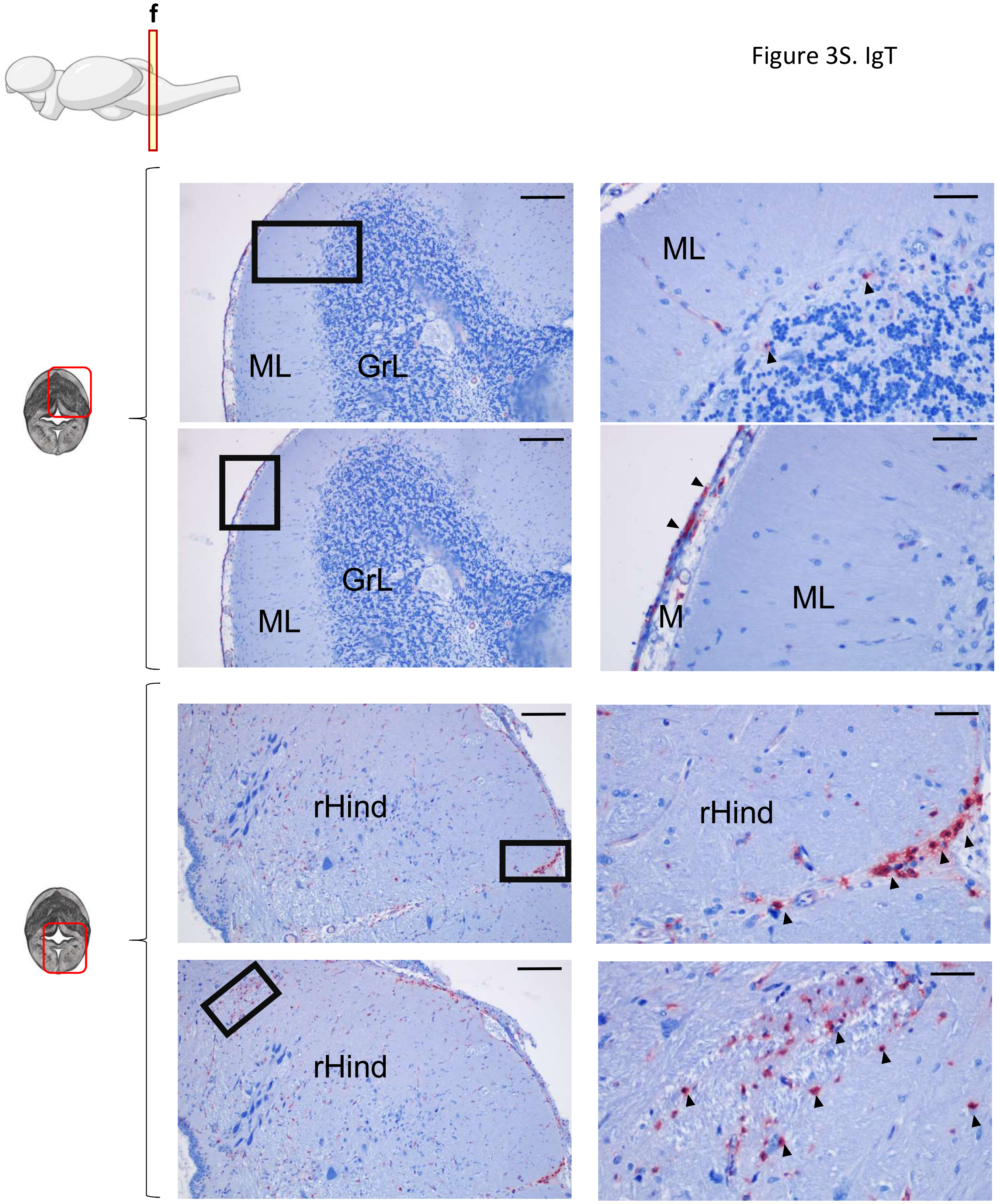

Figure S3. IgT^{+} B cells are also present in posterior part of rainbow trout cerebellum. Rainbow trout brains were sampled and processed as described in the Material and Methods section for immunohistochemical analysis of the posterior area of cerebellum (Cb) using a specific anti-trout IgT mAb. Representative images are shown (left panels) with black squares indicating the areas shown at higher magnification (right panels). Arrowheads indicate IgT^{+} B cells. Isotypes controls were included in all cases (not shown). A drawing of a rainbow trout brain indicating the location of the section analyzed is shown (top) along with drawings of the frontal views of each zone within analyzed Cb section. Molecular layer (ML), granular layer (GrL), meninx (M), rest of hindbrain (rHind). Scales bars: 200 µm (left images); 50 µm (right images).

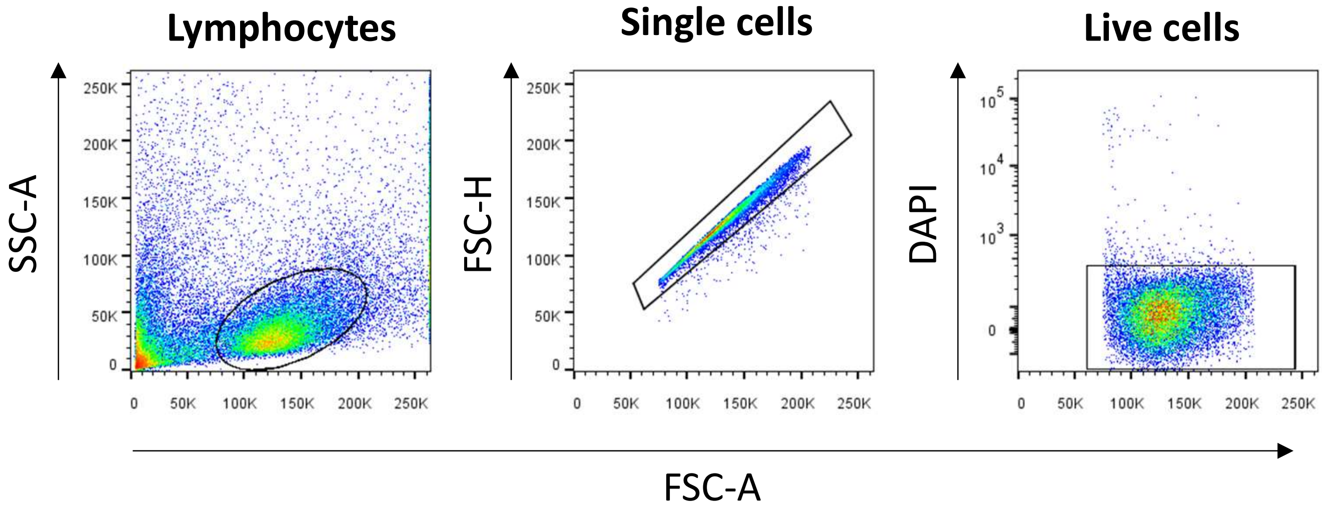

Figure S4. Gating strategy. Leukocytes were isolated from trout brain. FSC/SSC profile including a defined gate for lymphoid cells is shown. FSC-H/FSC-A profile within the lymphoid gate indicates singlets. DAPI negative cells within singlet gate were gated in order to select alive cells.

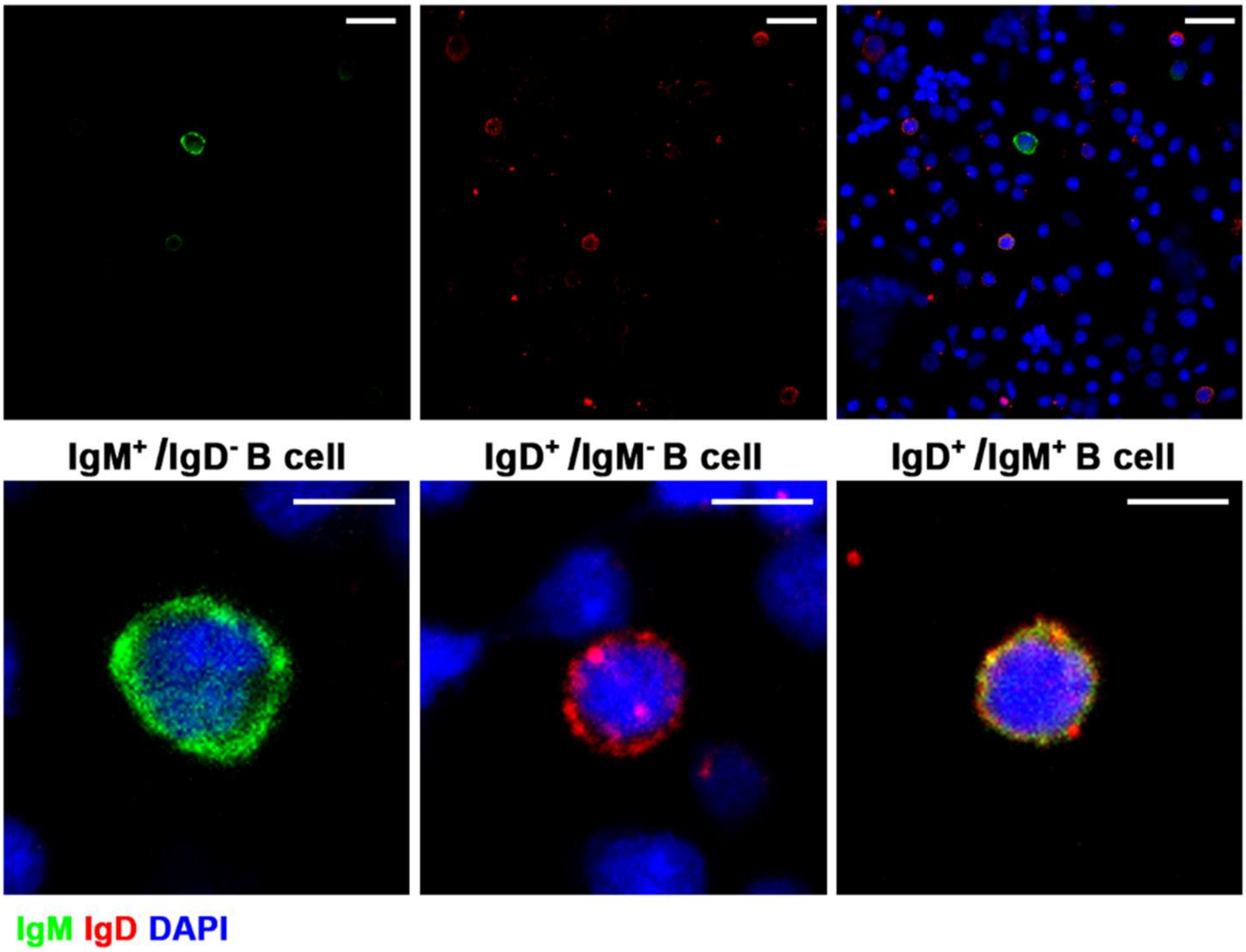

Figure S5. Double immunofluorescent detection of IgM and IgD in brain lymphocytes under the confocal microscope. Brain leukocytes were isolated as described in the Material and methods section for confocal microscope and stained with specific anti-trout IgD and anti-trout IgM mAbs for immunofluorescence analysis under the confocal microscope. All brain cell samples were counterstained with DAPI (1 µg/ml). Visualization of the different brain B cell subsets including IgM+IgD+, IgD+IgM+ and IgM+IgD+ cells was performed under the confocal microscope. Examples of each subset are shown in digital magnifications images. Scale bars: 20 µm (upper images); 5 µm (lower images).

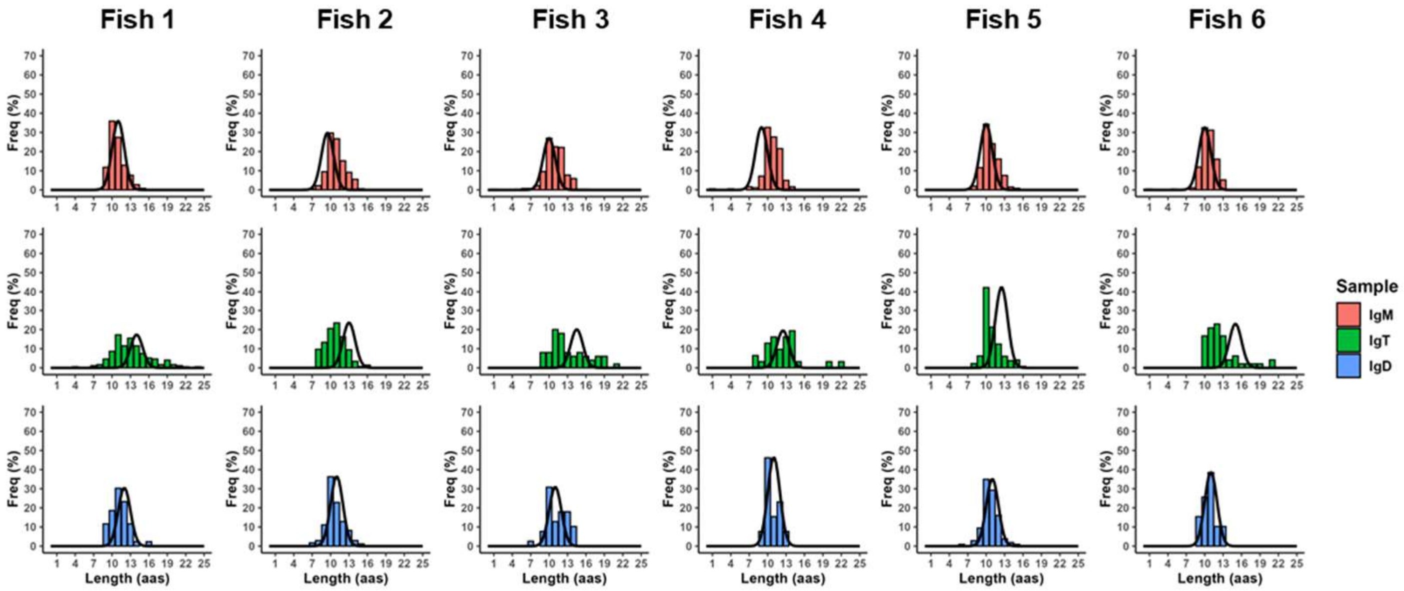

Figure S6. Clonal selection in IgH from brain samples. CDR3 spectrotyping from all JST sequences. The black curved lines represent the theoretical normal distribution adjusted to each plot. All graphs follow a non-normal distribution, as tested by Shapiro's assay.

Table S1. Primers used in different step of library construction for repertoire analysis

Primer	Sequence (5´- 3´)
cDNA synthesis	
oligodT	AAGCAGTGGTATCAACGCAGAGTACTTTTTTTTTTTTTTTTTTTTTTTTTTTTVN
TSO_UMI	CTACACGACGCUCTTCCGAUCTUNNNNUNNNNUNNNNUCTTrGrGrGrG*
Target Enrichment PCR 1	
Target_Enrichment_FW1	CTACACGACGCTCTTCCGATCT
IgM_R1	AAAGTCATTGGCAAAGCAGG
IgD_R1	ACCTCTGACCCTTGAGTCCA
IgT_R1	AGTCACCGTCAGTGGTTCTGT
Target Enrichment PCR 2	
Target_Enrichment_FW2	CTCTTTCCCTACACGACGCTC
IgM_&_IgD_R2	GTGACTGGAGTTCAGACGTGTGCTCTTCCGATCTCCAGAGTCATCATATCTCCGGT
IgT_R2	GTGACTGGAGTTCAGACGTGTGCTCTTCCGATCTATGTCGTTAGAAGGGGTTCCA
Index PCR	
P5_index	AATGATACGGCGACCACCGAGATCTACACXXXXXXXXACACTCTTTCCCTACACGACGCTC
P7_index	CAAGCAGAAGACGGCATACGAGATCGTGATXXXXXXXXGTGACTGGAGTTCAGACGTGT
V and N – IUPAC codes; rG – RNA base; U – DNA base	
